# Supplementary material for: Associations between food insecurity in high-income countries and pregnancy outcomes: A systematic review and meta-analysis
Source: PLoS Med. 2024 Sep 10;21(9):e1004450. doi: 10.1371/journal.pmed.1004450 (PMC11386426; doi:10.1371/journal.pmed.1004450)
Supplement: S1 Table — (DOCX) [file pmed.1004450.s002.docx]

**Table S1. Translation of search terms across databases**

| **Database** | **Search terms** |
| --- | --- |
| Ovid MEDLINE(R) 1946 to 21 November 2023 | 1. Pregnant Women/  2. exp Pregnancy/  3. pregnan*.mp.  4. matern*.mp.  5. gestat*.mp.  6. pre natal.mp.  7. prenatal.mp.  8. ante natal.mp.  9. antenatal.mp.  10. peri natal.mp.  11. perinatal.mp.  12. food insecur*.mp.  13. food secur*.mp.  14. (food adj2 insufficien*).mp.  15. (food adj2 sufficien*).mp.  16. food poverty.mp.  17. Poverty/  18. food.mp.  19. Food Deprivation/  20. (food adj2 depriv*).mp. [  21. exp Food Supply/  22. (food adj2 supply).mp.  23. food desert*.mp.  24. (food adj2 availab*).mp.  25. Hunger/  26. hunger.mp.  27. hungry.mp.  28. 1 or 2 or 3 or 4 or 5 or 6 or 7 or 8 or 9 or 10 or 11  29. 17 and 18  30. 12 or 13 or 14 or 15 or 16 or 19 or 20 or 21 or 22 or 23 or 24 or 25 or 26 or 27 or 29  31. Cross Section.mp.  32. Cross Section Stud*.mp.  33. Cross-sectional.mp.  34. Cross-sectional Stud*.mp.  35. Longitudinal Stud*.mp.  36. Longitudinal.mp.  37. "Case Control".mp.  38. Case Control Stud*.mp.  39. Case-Control.mp.  40. Case-Control Stud*.mp.  41. Retrospective Stud*.mp.  42. Retrospective.mp.  43. Cohort.mp.  44. Cohort stud*.mp.  45. Observational.mp.  46. Observational stud*.mp.  47. 31 or 32 or 33 or 34 or 35 or 36 or 37 or 38 or 39 or 40 or 41 or 42 or 43 or 44 or 45 or 46  48. 28 and 30 and 47  49. limit 48 to yr="2008- 2023"  50. limit 49 to humans |
| Ovid Embase 1974 to 21 November 2023 | 1. Pregnant Women/  2. exp Pregnancy/  3. pregnan*.mp.  4. matern*.mp.  5. gestat*.mp.  6. pre natal.mp.  7. prenatal.mp.  8. ante natal.mp.  9. antenatal.mp.  10. peri natal.mp.  11. perinatal.mp.  12. food insecur*.mp.  13. food secur*.mp.  14. (food adj2 insufficien*).mp.  15. (food adj2 sufficien*).mp.  16. food poverty.mp.  17. Poverty/  18. food.mp.  19. Food Deprivation/  20. (food adj2 depriv*).mp. [  21. exp Food Supply/  22. (food adj2 supply).mp.  23. food desert*.mp.  24. (food adj2 availab*).mp.  25. Hunger/  26. hunger.mp.  27. hungry.mp.  28. 1 or 2 or 3 or 4 or 5 or 6 or 7 or 8 or 9 or 10 or 11  29. 17 and 18  30. 12 or 13 or 14 or 15 or 16 or 19 or 20 or 21 or 22 or 23 or 24 or 25 or 26 or 27 or 29  31. Cross Section.mp.  32. Cross Section Stud*.mp.  33. Cross-sectional.mp.  34. Cross-sectional Stud*.mp.  35. Longitudinal Stud*.mp.  36. Longitudinal.mp.  37. "Case Control".mp.  38. Case Control Stud*.mp.  39. Case-Control.mp.  40. Case-Control Stud*.mp.  41. Retrospective Stud*.mp.  42. Retrospective.mp.  43. Cohort.mp.  44. Cohort stud*.mp.  45. Observational.mp.  46. Observational stud*.mp.  47. 31 or 32 or 33 or 34 or 35 or 36 or 37 or 38 or 39 or 40 or 41 or 42 or 43 or 44 or 45 or 46  48. 28 and 30 and 47  49. limit 48 to yr="2008- 2023"  50. limit 49 to humans |
| Ovid APA PsycInfo 1806 to 21 November 2023 | 1. Pregnant Women/  2. exp Pregnancy/  3. pregnan*.mp.  4. matern*.mp.  5. gestat*.mp.  6. pre natal.mp.  7. prenatal.mp.  8. ante natal.mp.  9. antenatal.mp.  10. peri natal.mp.  11. perinatal.mp.  12. food insecur*.mp.  13. food secur*.mp.  14. (food adj2 insufficien*).mp.  15. (food adj2 sufficien*).mp.  16. food poverty.mp.  17. Poverty/  18. food.mp.  19. Food Deprivation/  20. (food adj2 depriv*).mp. [  21. exp Food Supply/  22. (food adj2 supply).mp.  23. food desert*.mp.  24. (food adj2 availab*).mp.  25. Hunger/  26. hunger.mp.  27. hungry.mp.  28. 1 or 2 or 3 or 4 or 5 or 6 or 7 or 8 or 9 or 10 or 11  29. 17 and 18  30. 12 or 13 or 14 or 15 or 16 or 19 or 20 or 21 or 22 or 23 or 24 or 25 or 26 or 27 or 29  31. Cross Section.mp.  32. Cross Section Stud*.mp.  33. Cross-sectional.mp.  34. Cross-sectional Stud*.mp.  35. Longitudinal Stud*.mp.  36. Longitudinal.mp.  37. "Case Control".mp.  38. Case Control Stud*.mp.  39. Case-Control.mp.  40. Case-Control Stud*.mp.  41. Retrospective Stud*.mp.  42. Retrospective.mp.  43. Cohort.mp.  44. Cohort stud*.mp.  45. Observational.mp.  46. Observational stud*.mp.  47. 31 or 32 or 33 or 34 or 35 or 36 or 37 or 38 or 39 or 40 or 41 or 42 or 43 or 44 or 45 or 46  48. 28 and 30 and 47  49. limit 48 to yr="2008- 2023"  50. limit 49 to humans |
| Elsevier Scopus 2008 to 21 November 2023 | ( TITLE-ABS-KEY ( pregnan* OR matern* OR gestat* OR prenatal OR {pre natal} OR antenatal OR {ante natal} OR perinatal OR {peri natal} ) AND TITLE-ABS-KEY ( ( food AND insecur* ) OR ( food AND secur* ) OR ( food W/2 insufficien* ) OR ( food W/2 sufficien* ) OR ( food AND poverty ) OR ( food W/2 depriv* ) OR ( food W/2 supply ) OR ( food AND desert* ) OR ( food W/2 availab* ) OR hunger OR hungry ) AND TITLE-ABS-KEY (Case Control Stud* OR Case-Control Stud* OR Retrospective Stud* OR Cohort stud* OR Observational stud* OR longitudinal stud*) AND PUBYEAR > 2008  *Date: 2008-01-01 to 2023-21-11* |
| Clarivate Web of Science Core Collection 1970 to 21 November 2023 | TS=(pregnan* OR matern* OR gestat* OR prenatal OR “pre natal” OR antenatal OR “ante natal” OR perinatal OR “peri natal”) AND TS=(food insecur* OR food secur* OR (food NEAR/2 insufficien*) OR (food NEAR/2 sufficien*) OR food poverty OR (food NEAR/2 depriv*) OR (food NEAR/2 supply) OR food desert* OR (food NEAR/2 availab*) OR hunger OR hungry) AND TS=(Case Control Stud* OR Case-Control Stud* OR Retrospective Stud* OR Cohort stud* OR Observational stud* OR Longitudinal stud*) *Publication Date: 2008-01-01 to 2023-21-11* |
| ProQuest ASSIA 1987 to 21 November 2023 | (pregnan* OR matern* OR gestat* OR prenatal OR "pre natal" OR antenatal OR "ante natal" OR perinatal OR "peri natal") AND (food insecur* OR food secur* OR (food NEAR/2 insufficien*) OR (food NEAR/2 sufficien*) OR food poverty OR (food NEAR/2 depriv*) OR (food NEAR/2 supply) OR food desert* OR (food NEAR/2 availab*) OR hunger OR hungry) AND (case Control Stud* OR Case-Control Stud* OR Retrospective Stud* OR Cohort stud* OR Observational stud* OR longitudinal stud*) *Publication Date: 2008-01-01 to 2023-11-21* |
| EBSCO CINAHL 1981 to 21 November 2023 | ((MH "Pregnancy+") OR “pregnan*" OR "matern*" OR "gestat*" OR "prenatal" OR "pre natal" OR "antenatal" OR "ante natal" OR "perinatal" OR "peri natal") AND (“food insecur*” OR (MH "Food Security") OR “food secur*” OR “food N2 insufficien” OR “food N2 sufficien*” OR “food poverty” OR ((MH “Poverty”) AND “food”) OR “food N2 depriv*” OR (MH "Food Supply+") OR “food N2 supply” OR “food desert*” OR “food N2 availab*” OR (MH “Hunger”) OR “hunger” OR “hungry”) AND (“Case Control Stud”* OR “Case-Control Stud*” OR (MH “case control study+”) OR “Retrospective Stud*” OR (MH “retrospective study+”) OR “Cohort stud*” OR (MH “cohort study+”) OR “Observational stud*”) OR (MH “observational study+”) OR “longitudinal stud*” OR (MH “longitudinal study+”) *Publication Date: 2008-01-01 to 2023-11-21* |
| ProQuest Social Sciences Premium Collection (SSPC) 1981 to 21 November 2023 | (pregnan* OR matern* OR gestat* OR prenatal OR "pre natal" OR antenatal OR "ante natal" OR perinatal OR "peri natal") AND (food insecur* OR food secur* OR (food NEAR/2 insufficien*) OR (food NEAR/2 sufficien*) OR food poverty OR (food NEAR/2 depriv*) OR (food NEAR/2 supply) OR food desert* OR (food NEAR/2 availab*) OR hunger OR hungry) AND (case Control Stud* OR Case-Control Stud* OR Retrospective Stud* OR Cohort stud* OR Observational stud* OR longitudinal stud*) Publication Date: 2008-01-01 to 2023-11-21 |
