# Supplementary material for: Associations between food insecurity in high-income countries and pregnancy outcomes: A systematic review and meta-analysis
Source: PLoS Med. 2024 Sep 10;21(9):e1004450. doi: 10.1371/journal.pmed.1004450 (PMC11386426; doi:10.1371/journal.pmed.1004450)
Supplement: S2 Table — (DOCX) [file pmed.1004450.s003.docx]

**Table S2. Grey literature website searches**

| Name of website | URL | Date searched |
| --- | --- | --- |
| World Health Organization | <https://www.who.int/> | 30/01/2024 |
| The Trussell Trust (UK) | <https://www.trusselltrust.org/> | 30/01/2024 |
| Feeding Britain | <https://feedingbritain.org/> | 30/01/2024 |
| The Food Foundation (UK) | <https://foodfoundation.org.uk/> | 30/01/2024 |
| Feeding America | <https://www.feedingamerica.org/> | 30/01/2024 |
| Food Banks Canada | <https://foodbankscanada.ca/> | 30/01/2024 |
| Foodbank Australia | <https://www.foodbank.org.au/?state=au> | 30/01/2024 |
| The Foodbank Project (New Zealand) | <https://www.foodbank.org.nz/> | 30/01/2024 |
| European Food Banks Federation | <https://www.eurofoodbank.org/> | 30/01/2024 |
| Trove | https://trove.nla.gov.au/ | 30/01/2024 |
| Open Access Theses and Dissertations (OATD) | https://oatd.org/ | 30/01/2024 |
