# Supplementary material for: Associations between food insecurity in high-income countries and pregnancy outcomes: A systematic review and meta-analysis
Source: PLoS Med. 2024 Sep 10;21(9):e1004450. doi: 10.1371/journal.pmed.1004450 (PMC11386426; doi:10.1371/journal.pmed.1004450)
Supplement: S3 Table — (DOCX) [file pmed.1004450.s004.docx]

**Table S3. Details of contact with study authors**

| **Citation** | **Reason for contact** | **Author response** | **Decision** |
| --- | --- | --- | --- |
| Gordon and DeFranco, 2022 [1] | Full text (search only identified abstract) | Full text not published yet | Exclude – not reporting primary data |
| Willingham et al., 2020 [2] | Full text (search only identified abstract) | No reply | Exclude – not reporting primary data |
| Benes et al., 2020 [3] | Full text (search only identified abstract) | Full text not published yet | Exclude – not reporting primary data |
| Gordon and DeFranco, 2020 [4] | Full text (search only identified abstract) | Full text not published yet | Exclude – not reporting primary data |
| Crandall et al., 2020 [5] | Study date | Data collected in 2018 | Include |
| Sawanhkum et al., 2020 [6] | Full text (search only identified abstract) | Full text not published yet | Exclude – not reporting primary data |
| Chen et al., 2014 [7] | Full text (search only identified abstract) | No reply | Exclude – not reporting primary data |
| Steinmetz et al., 2020 [8] | Full text (search only identified abstract) | No reply | Exclude – not reporting primary data |
| Sandoval et al., 2020 [9] | Full text (search only identified abstract) | No reply | Exclude – not reporting primary data |
| Goodman et al., 2016 [10] | Full text (search only identified abstract) | Full text provided | Exclude – wrong exposure |
| Raghavan et al., 2019 [11] | Full text (search only identified abstract) | No reply | Exclude – not reporting primary data |
| Green et al., 2021 [12] | Full text (search only identified abstract) | No reply | Exclude – not reporting primary data |
| Yee et al., 2022 [13] | Full text (search only identified abstract) | Full text not published | Exclude – not reporting primary data |
| Patel et al., 2022 [14] | Full text (search only identified abstract) | No reply | Exclude – not reporting primary data |
| Menard et al., 2017 [15] | Full text (search only identified abstract) | No reply | Exclude – not reporting primary data |
| Villegas et al., 2017 [16] | Full text (search only identified abstract) | No reply | Exclude – not reporting primary data |
| Saleeby et al., 2017 [17] | Full text (search only identified abstract) | No reply | Exclude – not reporting primary data |
| Koleilat et al., 2010 [18] | Full text (search only identified abstract) | No reply | Exclude – not reporting primary data |
| Hromi-Fiedler et al., 2009 [19] | Full text (search only identified abstract) | Full text not published | Exclude – not reporting primary data |
| Chen et al., 2013 [20] | Full text (search only identified abstract) | No reply | Exclude – not reporting primary data |
| Cordeiro et al., 2013 [21] | Full text (search only identified abstract) | No reply | Exclude – not reporting primary data |
| Hromi-Fiedler et al., 2013 [22] | Full text (search only identified abstract) | Full text not published | Exclude – not reporting primary data |
| Power et al., 2017 [23] | Number data for graph | Initial response with no follow-up information provided | Include - (used the in-text figures combining both before and during pregnancy data) |
| Sullivan et al., 2021 [24] | Number data for pre-term birth outcome | No reply | Include - (used the data as reported) |
| Laraia et al., 2022 [25] | Number of women in each case for meta-analysis | Authors cannot access to the data | Include - No data for pooling in meta-analysis |
| Grilo et al., 2015 [26] | Request data for how many women with food insecurity (acute/ chronic) and with food security had depression (CES-D scale > 16), had anxiety, had low birth weight babies, and had preterm birth babies? | No reply | Include - No data provided |
| Cheu et al. 2020 [27] | Request mean (SD) data for birthweight and food insecurity statuses for meta-analysis | No reply (email is not delivered) | Include - No data provided |
| Joseph et al. 2023 [28] | Request frequency data for the different pregnancy outcomes by food insecurity statuses for meta-analysis | No reply (email is not delivered) | Include - No data provided |

**References**

1. Gordon A, Madzia J, DeFranco E. Assessing food insecurity's influence on large for gestational age for women residing in food deserts. American Journal of Obstetrics and Gynecology. 2020;222(1, Supplement):S131.<https://doi.org/10.1016/j.ajog.2019.11.202>.

2. Willingham L, Gordon J, Stokes E, Scott W, Fleenor R. Association between food deserts and pregnancy outcomes. American Journal of Obstetrics and Gynecology. 2020;222(1, Supplement):S265-S6.<https://doi.org/10.1016/j.ajog.2019.11.418>.

3. Benes LR, Yee LM, Levesque J, Miller ES. Is food insecurity during pregnancy associated with risk factors for maternal-to-child transmission of HIV? American Journal of Obstetrics and Gynecology. 2020;222(1, Supplement):S382-S3.<https://doi.org/10.1016/j.ajog.2019.11.612>.

4. Gordon A, Madzia J, DeFranco E. The impact of food insecurity on fetal growth restriction for women in Ohio food deserts. American Journal of Obstetrics and Gynecology. 2020;222(1, Supplement):S742-S3.<https://doi.org/10.1016/j.ajog.2019.11.1219>.

5. Crandall AK, Temple JL, Kong KL. The association of food insecurity with the relative reinforcing value of food, BMI, and gestational weight gain among pregnant women. Appetite. 2020;151:104685.<https://doi.org/10.1016/j.appet.2020.104685>.

6. Sawangkum P, Tanner J, Campos A, Fryer K, Wilson R, Louis-Jacques A. Does Food Access Impact Neonatal Birth Weight? Obstetrics & Gynecology. 2020;135

7. Chen S, Peterman J, Purdue-Smithe A, Otis N, Cordeiro L. Household food insecurity is associated with depression among young Cambodian women in Massachusetts. The FASEB Journal. 2014;28(S1):LB476.<https://doi.org/10.1096/fasebj.28.1_supplement.lb476>.

8. Steinmetz L, Hage M, Klavans M, Gambala C. Predictors of Gestational Diabetes Among Women Living in Food Deserts. Obstetrics & Gynecology. 2020;135

9. Sandoval V, Saleeby E, Jackson A, Smith L, Schickedancz A. Unmet Social Needs in the Prenatal Period: Effects on Birth Outcomes and Child Health. Obstetrics & Gynecology. 2020;135

10. Goodman MH, Thomson JL, Tussing-Humphreys LM. Diet Quality of a Pregnant Primarily African American Cohort Residing in the Mississippi Delta: Delta Healthy Sprouts. The FASEB Journal. 2016;30(S1):410.3-.3.<https://doi.org/10.1096/fasebj.30.1_supplement.410.3>.

11. Raghavan D, Haugsdal ML, Syrop CH. Upstream Obstetrics: Assessing the Prevalence of Food Insecurity in a High-Risk Obstetrics Population. Obstetrics & Gynecology. 2019;133

12. Green CA, Johnson JD, Vladutiu C, Manuck TA. Living in a county with high food insecurity is associated with preterm birth. American Journal of Obstetrics and Gynecology. 2021;224(2, Supplement):S331-S2.<https://doi.org/10.1016/j.ajog.2020.12.545>.

13. Yee LM, Niznik CM, DiTosto JD, Gomez Slagle HB, Ekpe EE, Summerlin S, et al. Associations of mindfulness, food security, and health literacy with diabetes-related perinatal health outcomes. American Journal of Obstetrics and Gynecology. 2022;226(1, Supplement):S709-S10.<https://doi.org/10.1016/j.ajog.2021.11.1171>.

14. Patel N, Cockerham C, Chilukuri P, Stanley Z, Vignes K, MacLeod EL, et al. Food & Housing Insecurity Influence on Outcomes in Pregnant Patients with Substance Use Disorder (SUD). American Journal of Obstetrics and Gynecology. 2022;226(1, Supplement):S280-S1.<https://doi.org/10.1016/j.ajog.2021.11.477>.

15. Menard V, Weiler H. Evaluation of pregnancy outcomes among food insecure women attending the Montreal Diet Dispensary program. The FASEB Journal. 2017;31(S1):961.1-.1.<https://doi.org/10.1096/fasebj.31.1_supplement.961.1>.

16. Villegas E, Hannon B, Hammons A, Teran-Garcia M, Wiley A. Is Food Security Influencing Emotional Health? A Study with Hispanic Immigrant Mothers. The FASEB Journal. 2017;31(S1):791.27-.27.<https://doi.org/10.1096/fasebj.31.1_supplement.791.27>.

17. Saleeby E, Scibetta E, Moini M, Trang C, Young CC, Greenwell L. MAMA'S Neighborhood–Maternity Assessment Management Access & Service Synergy Through the Neighborhood for Health. Obstetrics & Gynecology. 2017;129(5)

18. Koleilat M, Harrison G, Whaley S, Gomez J, Jenks E. Convenience Stores are Associated with Early Childhood Obesity in Low-Income Households. The FASEB Journal. 2010;24(S1):936.14-.14.<https://doi.org/10.1096/fasebj.24.1_supplement.936.14>.

19. Hromi-Fiedler A, Bermúdez-Millán A, Chapman D, Segura-Pérez S, Damio G, Melgar-Quiñonez H, et al. Food insecurity is associated with pregnancy outcomes among low-income Latinas. The FASEB Journal. 2009;23(S1):737.4-.4.<https://doi.org/10.1096/fasebj.23.1_supplement.737.4>.

20. Chen S, Peterman JN, Mouth R, Cordeiro L. Household Food Insecurity is Positively Associated with Perceived Discrimination among Cambodian Pregnant Women in Massachusetts. The FASEB Journal. 2013;27(S1):1054.18-.18.<https://doi.org/10.1096/fasebj.27.1_supplement.1054.18>.

21. Cordeiro LS, Peterman JN, Chen S, Mouth R, An R. Measuring the Food Security Status of Cambodian Women. The FASEB Journal. 2013;27(S1):369.1-.1.<https://doi.org/10.1096/fasebj.27.1_supplement.369.1>.

22. Hromi-Fiedler A, Bermúdez-Millán A, Leon J, Segura-Pérez S, Pérez-Escamilla R. Persistent food insecurity is associated with active coping strategies among low-income pregnant Latinas. The FASEB Journal. 2013;27(S1):1054.17-.17.<https://doi.org/10.1096/fasebj.27.1_supplement.1054.17>.

23. Power M, Uphoff E, Kelly B, Pickett KE. Food insecurity and mental health: an analysis of routine primary care data of pregnant women in the Born in Bradford cohort. J Epidemiol Community Health. 2017;71(4):324-8.10.1136/jech-2016-207799.

24. Sullivan K, St John M, DeFranco E, Kelly E. Food Insecurity in an Urban Pregnancy Cohort. Am J Perinatol. 2021;40(1):57-61.10.1055/s-0041-1729159.

25. Laraia BA, Gamba R, Saraiva C, Dove MS, Marchi K, Braveman P. Severe maternal hardships are associated with food insecurity among low-income/lower-income women during pregnancy: results from the 2012–2014 California maternal infant health assessment. BMC Pregnancy and Childbirth. 2022;22(1):138.10.1186/s12884-022-04464-x.

26. Grilo SA, Earnshaw VA, Lewis JB, Stasko EC, Magriples U, Tobin J, et al. Food Matters: Food Insecurity among Pregnant Adolescents and Infant Birth Outcomes. J Appl Res Child. 2015;6(2)

27. Cheu L, Yee L, Kominiarek M. Food insecurity during pregnancy and gestational weight gain. American journal of obstetrics and gynecology. 2020;220(1):204-.10.1016/j.ajog.2018.11.309.

28. Joseph NT, Stanhope KK, Geary F, McIntosh M, Platner MH, Wichmann HK, et al. Social Determinants of Health Needs and Perinatal Risk in Socially Vulnerable Pregnant Patients. J Health Care Poor Underserved. 2023;34(2):685-702.10.1353/hpu.2023.0058.
