# Supplementary material for: Associations between food insecurity in high-income countries and pregnancy outcomes: A systematic review and meta-analysis
Source: PLoS Med. 2024 Sep 10;21(9):e1004450. doi: 10.1371/journal.pmed.1004450 (PMC11386426; doi:10.1371/journal.pmed.1004450)
Supplement: S4 Table — (DOCX) [file pmed.1004450.s005.docx]

**Table S4. Adapted Newcastle-Ottawa Scale for Cohort Studies**

| **Selection**  Q1) Representativeness of the exposed cohort (i.e. those experiencing food insecurity during pregnancy) **– select one only** |
| --- |
| a) truly representative of the average population of pregnant women in the community (e.g. all women recruited in a set time period) * |
| b) somewhat representative of the average population of pregnant women in the community (e.g. they compared the recruited population with pregnant women in the local population and they have similar characteristics) ***** |
| c) selected group of users (e.g. those with gestational diabetes mellitus, aged over 30, restricted to one ethnic group, etc) |
| d) no description of the derivation of the cohort |
|  |
| Q2) Selection of the non-exposed cohort (i.e. those not experiencing food insecurity during pregnancy) **– select one only** |
| a) drawn from the same community as the exposed cohort (it will usually be this answer if they have recruited women from the same place at the same time) ***** |
| b) drawn from a different source (e.g. recruited women from the same place but at a different time, or from a different place) |
| c) no description of the derivation of the non-exposed cohort |
|  |
| Q3) Ascertainment of exposure **– select one only** |
| a) secure record (e.g. explicitly measured food insecurity - documented evidence from government benefits systems or equivalent etc) * |
| b) structured interview (i.e. validated self-report of food insecurity using a food insecurity scale/survey, and using the self-report measure in the analysis as the exposure variable) * |
| c) any self-report (not validated - e.g. person says they are food insecure without using a validated scale / definition) |
| d) no description |
|  |
| **Comparability** |
| Q4) Comparability of cohorts on the basis of the design or analysis - **a and b can both be selected** |
| a) study controls for pre-pregnancy body mass index ***** |
| b) study controls for any additional factor *****  (e.g. ethnicity) |
| c) no factors controlled for |
| both a) and b) |
|  |
| **Outcome** |
| Q5) Assessment of outcome **– select one only** |
| a) independent blind assessment (e.g. outcome specifically measured for the research) ***** |
| b) record linkage (e.g. outcomes from routine medical records) ***** |
| c) self-report |
| d) no description |
|  |
| Q6) Was follow-up long enough for outcomes to occur **– select one only** |
| a) yes (followed up for long enough for the outcome to develop, e.g. gestational diabetes mellitus assessed late in pregnancy) ***** |
| b) no |
|  |
| Q7) Adequacy of follow up of cohorts **– select one only** |
| a) complete follow up - all subjects accounted for (i.e. no loss to follow up for prospective cohorts, or for retrospective cohorts there is no missing data) * |
| b) subjects lost to follow-up unlikely to introduce bias - small number lost (>80% follow up for prospective cohorts, or for retrospective cohorts >80% with the data required for analysis), or description provided of those lost ***** |
| c) follow up rate < 80% for prospective cohorts, or >20% excluded due to missing data for the analysis for retrospective cohorts, and no description of those lost |
| d) no statement |
|  |
| Low (0-2 stars), Medium (3-5 stars), High (6-8 stars)  ^1^The maximum number of stars a study can be awarded has been reduced from 9 to 8 due to the removal of Question 4 from the original scale. The Original Question 4: “Demonstration that outcome of interest was not present at start of study” is a question relating to cohort studies looking at disease onset (e.g. prospective cohort exploring development of cancer of diabetes) where there should be checks that the participants do not already have these diseases at enrolment. This question is not applicable to GWG or dietary outcomes in the context of pregnancy and has therefore been removed from the scale. |
