# Supplementary material for: Associations between food insecurity in high-income countries and pregnancy outcomes: A systematic review and meta-analysis
Source: PLoS Med. 2024 Sep 10;21(9):e1004450. doi: 10.1371/journal.pmed.1004450 (PMC11386426; doi:10.1371/journal.pmed.1004450)
Supplement: S5 Table — (DOCX) [file pmed.1004450.s006.docx]

**Table S5a. Table of included studies additional characteristics**

| **First author, publication year** | **Study design** | | |
| --- | --- | --- | --- |
|  | **Participant inclusion criteria** | **Participant exclusion criteria** | **Outcomes: definition and measurement** |
| Richards et al., 2021 [1] | Currently pregnant, aged 18-49, completed two pregnancy visits. | If they were infertile, in prison or could not provide their informed consent, did not have any data from after 20 weeks, had a prior diagnosis of diabetes, if they were missing data on household FI, or pregnant with twins. | Gestational diabetes defined as glucose intolerance developed during pregnancy, assessed as self-report in 3^rd^ trimester or during a 26-week phone interview, or through medical charts at the end of pregnancy. |
| Luke. 2017 [2] | Singleton term pregnancies that lasted between 37–42 weeks. | Women younger than 18 years old or older than 45. | Level of exposure to stress over the last 12 months was measured by survey asking about the total number of stressful events. |
| Grilo et al., 2015 [3] | Aged 14-21, <24 weeks’ gestation, no indications of a high-risk pregnancy, English or Spanish speaking, willing to participate in a RCT. | Not reported. | LGA, SGA, gestational hypertension. |
| Mak. 2019 [4] | Adult women aged 18-49 pregnant at the time of their CCHS interview. | Individuals living on First Nations reserves and settlements in the provinces, full time members of the Canadian Armed Forces, institutionalized individuals including those living in prisons and care homes, and the Quebec health regions of Nunavik and Terres-Cries-de-la-Baie-James. Pregnant adolescents 15-17. | Birthweight-measured by trained research staff. |
| Cheng et al., 2022 [5] | Pregnant women receiving care at three community health centres that primarily serve low-income and racial/ethnic minority patients in Revere, Chelsea and Dorchester, MA. | Women who had a repeated record, or who had missing answers to FI questions. | Canadian Community Health survey (CCHS) from 2005-2014 measured mental health using four measures: fair/poor self-rated mental health, worse health compared to a year ago, weak sense of community, high life stress, a diagnosis of a mood disorder and a diagnosis of an anxiety disorder. Maternal hyperglycaemia were calculated using EMR data. |
| Mehta et al., 2020 [6] | Women between 8-23 weeks pregnant, 18-45 years old, English-speaking, annual household income up to 500% of the federal poverty line, and self-reported BMI between 25-40 kg/m2 (overweight and obese). | Women with PCOS and were treated with metformin, active substance abuse, recent weight loss, chronic corticosteroid use, pre-existing diabetes, an eating disorder, a positive early pregnancy diabetes screening test multiple gestation, or a history of gastric bypass surgery. | Maternal organohalogen measurements were measured from fasting maternal blood sample. |
| Eick et al., 2020 [7] | Pregnant women >18 years old, singleton pregnancy in the second trimester, speaking English or Spanish as their primary language. | Not reported. | Psychosocial stressors were measured using staff administered questionnaires: 4-item PSS, 10-item CES-D, The MacArtuhr Scale of Social Status for perceived community status. |
| Lairara et al., 2022 [8] | Women who are California residents, >15 years of age, singleton, twin or triplet birth during 2010-2012, household income <400% of the FPG, and have provided all household income information. | Women <15 years of age, non-residents and women with multiple births greater than three. Excluded from additional analyses: women who reported a racial/ethnic group other than White, Black, Latina, or Asian/Pacific Islander or had incomplete maternal hardship information. | Prenatal depressive symptoms was measured as part of the MIHA survey with questions focuses on severe maternal hardships including a question relating to experiencing depressive symptoms. |
| Goin et al., 2021 [9] | Women at Zuckerberg San Francisco General Hospital, Moffit Long Hospital, >18 years of age, with singleton pregnancies in the second trimester, and spoke English or Spanish as their primary language. | Not reported. | Birthweight and gestational age at birth were both abstracted from mother’s medical records which were linked to child’s medical records at birth. |
| Tarasuk et al. 2020 [10] | Women living in Ontario, who responded to any of the 4 linked cycles of the survey, had a valid Ontario health insurance plan number, delivered a live birth between 9 months prior and 6 months after the interview date. | Women with multiple pregnancies, those missing FI responses, any missing information on education or home ownership. | Medical record data from the Patient Health Questionnaire-2 (PHQ-2) and the Edinburgh Postnatal Depression Scale (EPDS). |
| Sandoval et al., 2021[11] | Low-income mother-child dyads in which the mother was screened by the MAMA’S Neighbourhood program and then later by paediatric clinic social needs screening between October 2018 and July 2019. | Not reported. | Anxiety score was measured using a 5-item tool developed by Rini et al. 1999. |
| Sullivan et al., 2021 [12] | Women who spoke proficient English and over 18 years of age, delivered at the University of Cincinnati Medical Centre between 2011 and 2019. | Women who could not speak proficient English to consent to survey and patients <18 years. | Resilience was measured by asking participants to rank the amount of joy/love/despair/hope they felt in their lives from 1 to 5, 1 being none and 5 being extreme amounts. |
| Power et al., 2017 [13] | Women 26-28 weeks pregnant between 2007-2010, who completed a health questionnaire, food insecurity survey, and had medical records 18 months prior to and 40 months after birth of cohort child. | Women <26 weeks or >28 weeks pregnant, outside of 2007-2010 and uncompleted records. | Perceived stress measured using the 4-item Perceived Stress Scale that produces a score on continuum. |
| Tucker et al., 2015 [14] | Women with a valid risk screening collected between August 31 2011 and May 20, 2012 with a corresponding delivery between Sep 1, 2011 and September 30, 2012. | Women were excluded if they were screened before 6 weeks or at or after 24 weeks gestation, if they had only Emergency Medicaid, or had a live birth prior to 24 weeks gestation. Women missing data on risk factors from the screening that could not be substituted with birth certificate data. | Depression measured using the 10-item CES-D questionnaire. |
| Richards et al., 2020 [15] | Women currently pregnant, or with the ability to become pregnant that were between 18 and 49 years old, and if women completed either of the two pregnancy visits. | Women who were infertile, in prison or could not provide informed consent, if no data was collected after 20 weeks, if they had prior diabetes or missing household food insecurity data or were pregnant with twins. | Community Status measured using the MacArthur of Social Status scale. |
| Cooper et al., 2022 [16] | Postpartum women who had prenatal care at the resident clinics at both UConn Health and Hartford Hospital and delivered term infants, defined as 37 weeks or longer gestation. | Women who had a preterm delivery, pre-gestational diabetes, an inability to obtain informed consent, and residence in a group home or rehabilitation centre. | Prenatal depressive symptoms self-reported. |
| Testa et al., 2022 [17] | Resident women who recently gave birth within their state to a live-born infant during the surveillance year. Women are sampled between 2 and 6 months after giving birth. | Not reported. | Fetal growth measured using birthweight for gestational age z-scores, which were calculated from a population reference to disentangle potential effects on gestational age versus fetal growth. |
| Cheu et al., 2020 [18] | Delivery of a live-born singleton neonate at ≥ 24 weeks, prenatal care recorded within the site’s electronic medical record, ≥18 years old, English speaking. | Pre-pregnancy/ predelivery BMI not available on the electronic medical record or if they declined to complete the entire survey. | Gestational diabetes, postpartum haemorrhage, preterm birth, mode of delivery; mood disorder; birthweight, LGA, SGA, 5-minute Apgar score, umbilical artery pH, shoulder dystocia, NICU admission and length of stay, neonatal hypoglycemia, and respiratory distress syndrome. |
| Duh-Leong et al, 2023 [19] | Infants between 1 and 21 days old, born at 34 weeks of gestation or later, weighing at least 2500 g or greater than 3rd percentile by World Health Organization curves and without a health condition affecting growth. | Parent-child dyads were excluded if the parent was not English or Spanish speaking or had uncorrected visual acuity problems. Analysis included the baseline assessment conducted in the first 21 days of the infant’s life, excluding participants who were not the birthing parent or had missing baseline data. | Gestational hypertension or diabetes measured using the Canadian Institute for Health Information Discharge Abstract Database. |
| Shriver et al., 2023 [20] | Maternal age ≥18 years; expecting a singleton birth; being fluent in oral and written English; planning to remain in the region for at least 3 years. | Not reported | GDM measurement not reported. |
| Eagleton et al., 2022 [21] | Maternal age ≥18; expecting a singleton birth, written English comprehension, and plans to remain in the region for at least 3 years. | No reported. | Depressive symptoms were measured using the 20-item CES-D scale. Anxiety symptoms were measured using The 20-item State Trait Anxiety Inventory. |
| Bihan et al., 2024 [22] | Singleton pregnancies, hyperglycemia in pregnancy. | Delivering twins or triplets, a personal history of diabetes, a history of bariatric surgery, no HIP screening, no healthcare cover, and missing EPICES questionnaire. | SGA, LGA were measured according to the standard for the French population. Clinical measures were used for hypertensive disorders, pre-eclampsia, caesarean section. Neonatal outcomes included shoulder dystocia, preterm birth, hypoglycaemia, neonatal death and stillbirth. |
| Oresnik, 2020 [23] | No description for the CCHS; For the (Mother to Babies (M2B) study): pregnant people living in the city of Hamilton. | Not reported. | CCHS survey asked questions regarding diagnosis of GDM, Mood disorder and Anxiety disorder. |
| Joseph et al., 2023 [24] | Women who attended prenatal care, completed at least one SDH screening questionnaire, and delivered one or more live or stillborn fetuses after 20-weeks’ gestation between October 2019–July 2021 at a large publicly supported hospital with 970 beds and approximately 2,500 deliveries per year. | Not reported. | Preeclampsia, GDM, Preterm birth (< 37 weeks), Caesarean delivery, Severe Maternal Morbidity, Stillbirth all obtained from EMRs. |
| Meeker et al., 2023 [25] | Data were analysed from 29 U.S. jurisdictions (Puerto Rico (births April–December 2020); Alaska, Connecticut, District of Columbia, Iowa, Maryland, Massachusetts, Missouri, Nebraska, Pennsylvania (Births June–December 2020); Arizona, Illinois, Louisiana, New Jersey, New York City, Oregon, South Dakota, Tennessee, Utah, Vermont, Virginia, West Virginia, Wyoming (births July–December 2020); Delaware (births August–December 2020); Arkansas, Georgia, Florida, Michigan, North Dakota (births October–December 2020) that implemented the PRAMS  COVID-19 Maternal Experiences supplement  administered October 2020–June 2021 among women with a live birth in April–December 2020, and achieved a response rate of 50% or higher" | Not reported. | Feeling more anxious than usual; Feeling more depressed than usual, measured using PRAMS. |

Abbreviation: BMI- Body Mass Index; CCHS – Canadian Community Health Survey; CES-D – Centre for Epidemiological Studies- Depression; EMR – Electronic Medical Records; FPG – Federal Poverty Guideline; GED – General Education Development; GDM – Gestational Diabetes Mellitus; HS – High School; MIHA – Maternal Infant Health Assessment; NICU- Neonatal Intensive Care Unit; PCOS – Polycystic Ovarian Syndrome; PRAMS – Pregnancy Risk Assessment Monitoring Systems; PSS – Perceived Stress Scale; IQR- Inter interquartile range.

**Table S5b. Table of included studies participant characteristics**

| **First author, publication year** | **Participants’ characteristics** | | | | |  |  |  |
| --- | --- | --- | --- | --- | --- | --- | --- | --- |
|  | **Age** | **Parity** | **Ethnic group** | **Income per annum** | **Education** | **Employment** | **Relationship status** | **Other socio-economic status indicators** |
| Richards et al., 2021 [1] | Median (IQR) 33 (25-33) | Not reported | White, Non-Hispanic: n=383 (64.7%);  Black, Non-Hispanic: n=35 (5.9%);  Hispanic: n=118 (19.9%);  Other: n=56 (9.5%) | <$10,000: n=70 (12.8%);  $10,000-$29,999: n=96 (17.6%);  $30,000-$49,999: n=92 (16.8%);  >$50,000: n=289 (52.8%) | Less than 12^th^ grade: n=79 (13.3%);  HS degree: n=218 (36.8%);  More than HS: n=295 (49.8%) | Not reported | Partnered: n=516 (87.2%);  Single 76 (12.8%) | Insurance  Private: n=355 (60.0%);  Medicaid: n=183 (30.9%);  Uninsured: n=54 (9.1%) |
| Luke. 2017 [2] | 18-24: n=2409 (25.2%);  25-29: n=2,943 (30.8%);  30-34: n=2,744(28.7%);  35+: n=1,548 (16.2%) | Previous live birth (Yes):  n= 5,563 (58.2%) | Non-Hispanic white: n=5,854 (61.2%);  Non-Hispanic black: n=363 (3.8%);  Hispanic: n=2,053 (21.5%);  Other: n=877 (9.2%) | Not reported | <HS: n=1,194 (12.5%);  HS: n=2,233 (23.4%);  >HS: n=6,217 (65.1%) | Not reported | Married: n=6,255 (65.5%) | WIC (yes):  n= 3,955 (41.4%) |
| Grilo et al., 2015 [3] | Mean (SD) 18.6 (1.7) | Nulliparous: n=735 (86.9%) | Latina: n=501 (56.9%);  Black, Non-Latina: n=308 (35.0%); White/other non-Latina: n=72 (8.2%) | Not reported | Enrolled in school  n=400 (45.6%) | Employed  n=194 (22.1%) | Single/never married: n=505 (58.9%);  Other: n=352 (41.1%) | Source of financial support  Self-support: n=284 (32.3%);  Other: n=594 (67.7%)  Housing insecure  n=232 (26.9%) |
| Mak. 2019 [4] | 18-24 years: n=771 (16%);  25-29 years: n=1,590 (33%);  30-34 years: n=1,493 (31%);  35-49 years: n=963 (20%) | Presence of children  No: n=2,071 (43%);  Yes: n=2,746 (57%) | Indigenous  No: n=4,624 (96%);  Yes: n=207 (4.3%) | Household Income Quintile  Quintile 1 (<$40,000) Quintile 2 ($40,000-$64,999)  Quintile 3 ($65,000-$89,999)  Quintile 4 ($90,000-$119,999)  Quintile 5 (≥$120,000)  Each quintile n=963 (20%) | Post-Secondary Education  No: n=1,252 (26%);  Yes: n=3,565 (74%) | Main Source of Household  Income  Wages, Salaries, Self-Employment: n=4,528 (94%);  Social Assistance: n=149 (3.1%);  Other: n=135 2.8% | Presence of a partner  No: n=578 (12%);  Yes: n=4,239 (88%) | Home ownership  No: n=1,541 (32%);  Yes: n=3,276 (68%) |
| Cheng et al., 2022 [5] | Mean (SD) 29.1 (5.8) | Not reported | White:  n=241 (28.1%);  Black of African American:  n=88 (10.3%);  Asian or others:  n=99 (11.6);  Hispanic or Latino:  n=429 (50.1%) | <$10,000:  n=127 (16.4%);  $10,001 to $20,000:  n=184 (23.7%);  $20,001 to $50,000  n=300 (38.7%);  >$50,000:  n=164 (21.2%) | Some HS or less:  n=151 (22.0%);  HS graduate:  n=256 (37.4%);  More than HS or other:  n=278 (40.6%) | Employment  Employed full time:  n=321 (37.6%);  Employed part time:  n=238 (27.9%);  Unemployed:  n=295 (34.5%) | Married/Living together:  n=689 (80.5%);  Unmarried:  n=167 (19.5%) | Not reported |
| Mehta et al., 2020 [6] | <=27 at enrolment:  n=51 (52.04%);  >27 at enrolment:  n=47 (47.96%) | Nulliparous:  n=46 (46.94%);  Multiparous:  n=52 (53.06) | White/Other:  n=33 (33.67%);  African American:  n=32 (32.65%);  Latina:  n=33 (33.67%) | <= 2011 poverty level:  n=44 (47.83%);  >2011 poverty level:  n=48 (52.17%) | <=HS graduate:  n=34 (34.69%);  >HS graduate:  n=64 (65.31%) | Not reported | Single or other status:  n=33 (33.67%);  In-relationship:  n=65 (66.33%) | Not reported |
| Eick et al., 2020 [7] | Mean (SD) 32 (5.4);  Missing:  n=1 (0.2%) | One or more prior births:  n=247 (48%);  Missing:  n=8 (1.6%) | Non-Hispanic White: n=194 (38%);  Non-Hispanic Black:  n=41 (8.0%);  Hispanic:  n=174 (34%);  Asian/Pacific Islander: n=95 (19%);  Missing:  n=6 (1.2%) | Not reported | Less than HS:  n=59 (12%);  HS degree/some college:  n=140 (27%);  College degree:  n=118 (23%);  Graduate degree:n=185 (36%);  Missing: n=8 (1.6%) | Not reported | Married: n=337 (66%);  Single: n=161 (32%);  Missing: n=12 (2.4%) | Not reported |
| Lairara et al., 2022 [8] | 15-19:  n=1280 (9.0%);  20-24:  n=3784 (25.5%);  25-29:  n=4253 (30.0%);  30-34:  n=3073 (21.5%);  35+:  n=1883 (13.2%) | Not reported | White:  n=4013 (28.1%);  Black:  n=1357 (9.5%);  Latina foreign-born:  n=4009 (28.1%);  Latina US-born:  n=3695 (25.9%);  Asian/Pacific Islander:  n=1199 (8.4%) | 0-100% FPG:  n=7927 (55.6%);  101-200% FPG:  n=3713 (26.0%);  201-300% FPG:  n=1503 (10.5%);  301-400% FPG:  n=1085 (7.6%) | <HS:  n=3141 (22.0%);  HS/Graduate education:  n=3664 (25.7%);  Some college:  n=5174 (36.3%);  College/graduate+:  n=2294 (16.1%) | Not reported | Married:  n=6773 (47.5%);  Living together:  n=4472 (31.3%);  Single:  n=3028 (21.2%) | Number of people living in Household  <2:  n=5184 (36.3%);  3-4:  n=6966 (48.8%);  >5:  n=2123 (14.9%) |
| Goin et al., 2021 [9] | Mean (SD) 32.4 (5.4) | Parity  0:  n=251 (49%);  1:  n=155 (30%);  2:  n=73 (14%);  3+:  n=32 (6%) | Latina:  n=175 (34%);  Asian or Pacific Islander:  n=86 (17%);  Black  n=37 (7%);  White  n=186 (37%);  Other or multiple  n=25 (5%) | < $40,000:  n=194 (38%);  $40,000-$79,000:  n=66 (13%);  >$80,000:  n=251 (49%) | Not reported | Not reported | Married:  n=343 (67%);  Widowed, separated, or divorced:  n=25 (5%);  Never married: n=141 (28%) | Not reported |
| Tarasuk et al. 2020 [10] | Mean (SD) 29.59 (5.38) | Previous live births  None:  n=937 (47.1%);  ≥1:  n=1051 (52.9%) | Non-White  n=410 (20.6%);  White  n=1578 (79.4%) | Main source of household income  Wages, salaries, self-employment:  n=1768 (88.9%)  Social assistance:  n= 92 (4.6%)  Other:  n=128 (6.4%) | <Secondary school graduation:  N=172 (8.7%);  Secondary graduate:  n=297 (14.9%);  Some post–secondary school:  n=112 (5.6%);  Post–secondary school graduate:  n=1407 (70.8%) | Not reported | Not reported | Housing tenure  Owner:  n=1402 (70.5%);  Renter:  n=586 (29.5%) |
| Sandoval et al., 2021[11] | Not reported | Number of previous live births:  Mean 0.87 (SD 1.16) | Hispanic or Latino:  n=185 (69.0%);  Not Hispanic or Latino:  n=83 (31.0%) | Not reported | Did not graduate HS:  n=77 (28.7%);  HS/GED:  n=144 (53.7%);  Some college or above:  n=47 (17.5%) | Employment status  Unemployed:  n= 135 (50.6%); Employed:  n=132 (49.4%) | Not reported | Not reported |
| Sullivan et al., 2021 [12] | <18:  n=9 (2.1%);  18-34:  n=380 (89.2%);  Over 35:  n=37 (8.6%) | >5 living children:  n=42 (9.9%) | non-Hispanic black women:  n=227 (53.0%);  non-Hispanic white women:  n= 170 (39.0%);  Hispanic women:  n=12 (3.8%);  Other:  n=17 (4%) | <$40,000: n=328 (76.9%) | No HS diploma n=115 (27.0%);  HS diploma/GED n=258 (60.6%);  Bachelor's degree or more:  n=53 (12.4%) | Employment  Unemployed: n=165 (38.7%) | Unmarried:  n=312 (72.7%) | Social security  WIC:  n=282 (66.2%);  Food Stamps: n=242 (56.8%);  Medicaid: n=284 (66.7%) |
| Power et al., 2017 [13] | Mean (SD) 27.66 (5.6) | Not reported | White British:  n=480 (37.5) ;  Pakistani:  n=624 (48. 8%);  Indian:  n=56 (4.4%);  Bangladeshi:  n=24 (1.9%);  White Other:  n=26 (2.0%);  Black:  n=25 (2.0%);  Other:  n=44 (3.4%) | Not reported | Not reported | Not reported | Not reported | Means-tested benefit receipt: Yes  n=646 (40.6%);  No  n=947 (59.5%) |
| Tucker et al., 2015 [14] | <18:  n=1,598 (10.36%);  19–34: n=13,027 (84.44%);  35 or over: n=802 (5.20%) | Nulliparous:  Yes:  n=8,831 (57.24%);  No:  n=6,597 (42.76) | Non-Hispanic white: n=7,154 (46.37%);  Non-Hispanic black: n=6,211 (40.26%); Asian/Pacific Islander: n=382 (2.48%); American Indian/Alaska Native: n=437 (2.83%); Hispanic:  n=802 (8.06%) | Not reported | HS: n=3,924 (25.40%);  HS graduate or GED:  n=5,478 (35.51%);  Some college or more:  n=6,022 (39.03%); Missing: n = 8 (0.05%) | Not reported | Married at conception or birth  No: n=10,942 (70.92%);  Yes: n= 4,473 (28.99%); Missing: n =14 (0.09%) | Medicaid for pregnant women  Yes: n=10,159 (65.85%);  No: n=5,269 (34.15%) |
| Richards et al., 2020 [15] | Mean (SD) 28.9 (5.6) | First pregnancy: n=182 (24.6%) | White, Non Hispanic: n=482 (64.6%);  Black, Non Hispanic: n=41 (5.5%);  Hispanic:  n=152 (20.4%);  Other:  n=71 (9.5%) | < $20,000: n=136 (19.9%);  $20,000 - $74,999: n=312 (45.6%);  > $75,000: n=237 (34.6%) | Less than 12th grade:  n=99 (13.3%);  HS degree:  n=256 (34.3%);  More than HS:  n=382 (51.2%) | Employment  Yes: n=432 (57.9%) | Partnered:  n=654 (87.7%) | 2 or more adults in household: n=549 (73.6%)  Insurance  Private: n=454 (60.9%);  Medicaid: n=224 (30.0%);  Uninsured:  n=68 (9.1%) |
| Cooper et al., 2022[16] | Mean (SD) 31.27 (6.54) | Multiparity  No:  n=22 (31%);  Yes:  n=48 (69%) | Caucasian:  n=27 (39%);  Black:  n=18 (26%);  Hispanic:  n=25 (36%) | Not reported | Not reported | Not reported | Not reported | Not reported |
| Testa et al., 2022 [17] | < 18:  n=169 (0.8%);  18-24:  n=4,195 (19.9%);  25-29:  n=6,429 (30.5%);  30-34:  n=6,556 (31.1%);  ≥ 35:  n=3,731 (17.7%) | 0: n=8,116 (38.5%);  1:  n=6,809 (32.3%);  2:  n=3,541 (16.8%);  3:  n=2,614 (12.4%) | White:  n=13,217 (62.7%);  Hispanic:  n=2,867 (13.6%);  Black:  n=3,394 (16.1%);  Other race or ethnicity:  n=1,602 (7.6%) | Household Income  ≤ $16,000 n=3,815 (18.1%);  $16,000-$40,000:  n=5,017 (23.8%);  $40,001-$85,000:  n=6,830 (32.4%);  > $85,000: n=5,418 (25.7%) | < HS  n=1,939 (9.2%);  HS graduate:  n=4,848 (23.0%);  Some college:  n=6,303 (29.9%);  College graduate: n=7,989 (37.9%) | Not reported | Married:  n=13,618 (64.6%) | Dental Insurance  No: n=4,406 (20.9%) |
| Cheu et al., 2020 [18] | Median (IQR)  Inadequate FI: 28 (26, 35)  Adequate FS: 33 (30, 36) | Primiparous:  n=166 (55.5%) | Non-Hispanic white:  n=67 (55.9%);  Non-Hispanic black:  n=53 (17.7%);  Hispanic:  n=6 (12.0%);  Asian or other:  n=43 (14.4%) | Not reported | Some college education or greater: n=274 (91.6%) | Employment  Yes n=236 (78.9%) | Married: n=223 (74.6%) | Public insurance  Yes: n=57 (19.1%) |
| Duh-Leong et al, 2023 [19] | Median (IQR)  30.1 (22.1–37.7) | Not reported. | Hispanic:  n=327 (41.6%);  White:  n=178 (22.6%);  Black  n=131 (16.6%);  Asian/Multi-Race/Other  n=151 (19.2%)  Country or origin  Born outside United States:  n=444 (56.4%);  Born in Mexico  n=118 (15.0%);  Born in Honduras  n=31 (3.9%);  Born in Ecuador n= 39 (5.0%) | < $20,000:  n=190 (24.1%);  $20,000–$49,999:  n=205 (26.0%) ;  $50,000–$99,999  n=96 (12.2%);  $100,000 or more  n=119 (15.1%);  Don’t know/Not sure  n=177 (22.5%) | Completed HS:  n=656 (83.4%) | Not reported | Married or Living as Married  n=546 (69.4%) | Not reported |
| Shriver et al., 2023 [20] | Mean (SD) 29.71 (5.48) | Not reported | Non-Hispanic White:  n=157 (52.5%);  Non-Hispanic Black:  n=86 (28.8%);  Hispanic/Other:  n=56 (18.7%) | Income to needs ratio:  Mean (SD) 3.49 (2.96) | <HS diploma /GED:  n=47 (15.9%);  Some college:  n=57 (19.3%);  2-year college degree:  n=24 (8.1%);  4-year college degree:  n=74 (25.1%);  Postgraduate work / degree:  n=93 (31.5%) | Not reported | Not reported | Not reported |
| Eagleton et al., 2022 [21] | Mean (SD) 29.04 (5.93) | Not reported | Race  White:  n=82 (51.2%);  Black:  n=55 (34.4%);  Other, biracial, and multiracial:  n=23 (14.3%)  Ethnicity  Hispanic:  n=14 (8.4%);  Non-Hispanic:  n=152 (91.6%) | Income-to-needs ratio: Mean (SD) 3.15 (3.01) | ≤HS diploma or GED:  n=36 (21.7%); Some college:  n=36 (21.7%);  2-year college degree:  n=17 (10.2%);  4-year college degree:  n=35 (21.1%); Postgraduate work or degree:  n=42 (25.3%) | Not reported | Not reported | Not reported |
| Bihan et al., 2024 [22] | Mean (SD) 32.7 ( 5.4) | Mean (SD) 2.3 (1.3) | Region of origin  Sub-Saharan Africa:  n = 179 (15.4%);  North Africa:  mean = 420 (36.0%);  Other:  n = 88 (7.5%);  Europe:  n = 225 (19.3%);  Haiti, French overseas territories:  n = 52 (4.5%);  South Asia:  n = 202 (17.3%) | Not reported | Not reported | Employment Working:  n = 427 (36.6%) | Not reported | Health insurance coverage  Social security:  n=444 (56.4%); Universal health protection:  n=190 (23.8%);  Complementary universal health protection:  n=93 (11.6%);  State medical aid:  n=72 (9.0%) |
| Oresnik, 2020 [23] | **M2B study**  15-19:  n=10 (3.07%);  20-24:  n=32 (9.72%);  25-29:  n=90 (27.37%);  30-34:  n=128 (38.62%); 35-39:  n=54 (16.37%); 40-44:  n=15 (4.60%); 45+:  n=1 (0.26%)  **CCHS 2017/18**  15-19:  n=78 (2.38%);  20-24:  n=325 (9.95%); 25-29:  n=873 (26.76%); 30-34:  n=984 (30.16%); 35-39:  n=578 (17.72%); 40-44:  n=223 (6.84%); 45+:  n=202 (6.20%) | Not reported | **M2B study**  White  n=243 (73.64%); Racialized group  n=87 (26.36%)  **CCHS 2017/18**  White:  n=2,255 (69.12%); Racialized group  n=1,007 (30.88%) | **M2B study**  <$23,000  n=57 (17.19%); $ 23,000-39,999  n=47 (14.33%); $ 40,000-79,999  n= (21.49%);  $ 80,000 +  n=155 (46.99%)  **CCHS 2017/18**  <$20,000  n=318 (9.75%);  $ 20,000-39,999  n=375(11.51%);  $ 40,000-59,999  n=441 (13.53%); $60,000-79,999  n=437 (13.38%); $80,000-99,999  n=346 (10.61%); $100,000 +  n=1,345 (41.22%) | **M2B study**  < HS: n=25 (7.44%);  HS: n=23 (6.92%); Some Post-Secondary: n=32 (9.74%);  Post-Secondary: n=250 (75.90%)  **CCHS 2017/18**  < HS: n=239 (7.34%);  HS: n=493 (15.10%);  Post-Secondary:  n=2,530 (77.56%) | **M2B study**  Employed: n=169 (51.15%); Unemployed:  n=161 (48.85%)  **CCHS 2017/18**  Employed: n=2,263 (69.36%); Unemployed: n=999 (30.64%) | **M2B study**  Married or Common  Law:  n=276 (83.72%);  Not Married or  Common Law: n=54 (16.28%).  **CCHS 2017/18**  Married or Common  Law: n=2,860 (87.68%);  Not Married or  Common Law: n=376 (12.32%) | Not reported |
| Joseph et al., 2023 [24] | Mean (SD) 27.7 (6.4) | Not reported | Hispanic:  n=190 (12.1%);  Non-Hispanic White: n=33 (2.1%);  Non-Hispanic Black: n=1294 (82.2%);  Non-Hispanic Other:  n=57 (3.6%) | Household median income  ($USD): Mean (SD) 49,478 (16,760)  % Households below Federal  Poverty Level: Mean (SD) 23.1 (8.3) | Not reported |  | Not reported | Insurance  Uninsured:  n=167 (10.5%); Public:  n=1395 (87.6%); Private:  n=31 (2%)  Average household size Mean (SD) 2.7 (0.3)  Neighbourhood deprivation  Mean (SD) 0.4 (1)  % Households without vehicle Mean (SD) 15.1 (8.8) |
| Meeker et al., 2023 [25] | <20:  n=4% (95% CI 3–4);  20–24:  n=18% (95% CI17–19);  25–34:  n=58% (95% CI 57–59);  ≥35:  n=21% (95% CI 20–22) | Not reported | Non-Hispanic White:  n=55% (% (95% CI 53–56);  Non-Hispanic Black: n=15% (% (95% CI 14–16);  Non-Hispanic American Indian/Alaska Native:  n=1% (% (95% CI 0–1);  Non-Hispanic Asian/Pacific Islander:  n=6% (% (95% CI 5–6);  Non-Hispanic other/persons of multiple races:  n=3% (% (95% CI 2–3);  Hispanic:  n=21% (% (95% CI 20–22) | Not reported | ≤HS: 37% (95% CI 35, 38);  >HS: 63% (95% CI 62, 65) | Not reported | Not reported | Delivery health insurance  Private: 54% (95% CI 52, 55);  Medicaid: 42%(95% CI 40, 43);  Other:  2% (95% CI 1–2);  None:  n=3 (95% CI 3–4) |

Abbreviation: BMI- Body Mass Index; CCHS – Canadian Community Health Survey; CES-D – Centre for Epidemiological Studies- Depression; EMR – Electronic Medical Records; FPG – Federal Poverty Guideline; GED – General Education Development; GDM – Gestational Diabetes Mellitus; HS – High School; MIHA – Maternal Infant Health Assessment; NICU- Neonatal Intensive Care Unit; PCOS – Polycystic Ovarian Syndrome; PRAMS – Pregnancy Risk Assessment Monitoring Systems; PSS – Perceived Stress Scale; IQR- Inter interquartile range.

**References**

1. Richards M, Weigel M, Li M, Rosenberg M, Ludema C. Food insecurity, gestational weight gain and gestational diabetes in the National Children's Study, 2009-2014. J Public Health (Oxf). 2021;43(3):558-66.10.1093/pubmed/fdaa093.

2. Luke S. Neighborhood deprivation, food insecurity and gestational weight gain.: University of South Florida; 2017.

3. Grilo SA, Earnshaw VA, Lewis JB, Stasko EC, Magriples U, Tobin J, et al. Food Matters: Food Insecurity among Pregnant Adolescents and Infant Birth Outcomes. J Appl Res Child. 2015;6(2)

4. Mak J. Food Insecurity During Pregnancy in Canada: University of Toronto; 2019.

5. Cheng ER, Luo M, Perkins M, Blake-Lamb T, Kotelchuck M, Arauz Boudreau A, et al. Household food insecurity is associated with obesogenic health behaviours among a low-income cohort of pregnant women in Boston, MA. Public Health Nutrition. 2022:1-9.10.1017/S1368980022000714.

6. Mehta SS, Applebaum KM, James-Todd T, Coleman-Phox K, Adler N, Laraia B, et al. Associations between sociodemographic characteristics and exposures to PBDEs, OH-PBDEs, PCBs, and PFASs in a diverse, overweight population of pregnant women. J Expo Sci Environ Epidemiol. 2020;30(1):42-55.10.1038/s41370-019-0173-y.

7. Eick SM, Goin DE, Izano MA, Cushing L, DeMicco E, Padula AM, et al. Relationships between psychosocial stressors among pregnant women in San Francisco: A path analysis. PLoS One. 2020;15(6):e0234579.10.1371/journal.pone.0234579.

8. Laraia BA, Gamba R, Saraiva C, Dove MS, Marchi K, Braveman P. Severe maternal hardships are associated with food insecurity among low-income/lower-income women during pregnancy: results from the 2012–2014 California maternal infant health assessment. BMC Pregnancy and Childbirth. 2022;22(1):138.10.1186/s12884-022-04464-x.

9. Goin DE, Izano MA, Eick SM, Padula AM, DeMicco E, Woodruff TJ, et al. Maternal Experience of Multiple Hardships and Fetal Growth: Extending Environmental Mixtures Methodology to Social Exposures. Epidemiology. 2021;32(1):18-26.10.1097/ede.0000000000001272.

10. Tarasuk V, Gundersen C, Wang X, Roth DE, Urquia ML. Maternal Food Insecurity is Positively Associated with Postpartum Mental Disorders in Ontario, Canada. J Nutr. 2020;150(11):3033-40.10.1093/jn/nxaa240.

11. Sandoval VS, Jackson A, Saleeby E, Smith L, Schickedanz A. Associations Between Prenatal Food Insecurity and Prematurity, Pediatric Health Care Utilization, and Postnatal Social Needs. Acad Pediatr. 2021;21(3):455-61.10.1016/j.acap.2020.11.020.

12. Sullivan K, St John M, DeFranco E, Kelly E. Food Insecurity in an Urban Pregnancy Cohort. Am J Perinatol. 2021;40(1):57-61.10.1055/s-0041-1729159.

13. Power M, Uphoff E, Kelly B, Pickett KE. Food insecurity and mental health: an analysis of routine primary care data of pregnant women in the Born in Bradford cohort. J Epidemiol Community Health. 2017;71(4):324-8.10.1136/jech-2016-207799.

14. Tucker CM, Berrien K, Menard MK, Herring AH, Daniels J, Rowley DL, et al. Predicting Preterm Birth Among Women Screened by North Carolina's Pregnancy Medical Home Program. Matern Child Health J. 2015;19(11):2438-52.10.1007/s10995-015-1763-5.

15. Richards M, Weigel M, Li M, Rosenberg M, Ludema C. Household food insecurity and antepartum depression in the National Children's Study. Ann Epidemiol. 2020;44:38-44.e1.10.1016/j.annepidem.2020.01.010.

16. Cooper S, Graham M, Kuo CL, Khangura R, Schmidt A, Bakaysa S. The Relationship between Food Security and Gestational Diabetes among Pregnant Women. AJP Reports. 2022;12(3):E131-E8.doi:10.1055/s-0042-1751082.

17. Testa A, Ganson KT, Jackson DB, Bojorquez-Ramirez P, Weiser SD, Nagata JM. Food insecurity and oral health care experiences during pregnancy: Findings from the Pregnancy Risk Assessment Monitoring System. J Am Dent Assoc. 2022;153(6):503-10.10.1016/j.adaj.2021.12.010.

18. Cheu L, Yee L, Kominiarek M. Food insecurity during pregnancy and gestational weight gain. American journal of obstetrics and gynecology. 2020;220(1):204-.10.1016/j.ajog.2018.11.309.

19. Duh-Leong C, Perrin EM, Heerman WJ, Schildcrout JS, Wallace S, Mendelsohn AL, et al. Prenatal Risks to Healthy Food Access and High Birthweight Outcomes. Acad Pediatr. 2023.10.1016/j.acap.2023.08.017.

20. Shriver LH, Eagleton SG, Hosseinzadeh M, Buehler C, Wideman L, Leerkes EM. Associations among eating behaviors, food security status, and dietary intake during pregnancy. Appetite. 2023;191:107062.<https://doi.org/10.1016/j.appet.2023.107062>.

21. Eagleton SG, Shriver LH, Buehler C, Wideman L, Leerkes EM. Longitudinal Associations Among Food Insecurity During Pregnancy, Parental Mental Health Symptoms, Controlling Feeding Styles, and Infant Food Responsiveness. The Journal of Nutrition. 2022;152(12):2659-68.10.1093/jn/nxac225.

22. Bihan H, Nachtargeale C, Vicaud E, Sal M, Berkane N, Pinto S, et al. Impact of experiencing multiple vulnerabilities on fetal growth and complications in women with hyperglycemia in pregnancy. BMC Pregnancy Childbirth. 2023;23(1):740.10.1186/s12884-023-06048-9.

23. Orsenik S. The Intersection of Food Insecurity, Gestational Diabetes and Mental Health Conditions: Examining Pregnancy from a Biocultural Perspective: McCaster University; 2020.

24. Joseph NT, Stanhope KK, Geary F, McIntosh M, Platner MH, Wichmann HK, et al. Social Determinants of Health Needs and Perinatal Risk in Socially Vulnerable Pregnant Patients. J Health Care Poor Underserved. 2023;34(2):685-702.10.1353/hpu.2023.0058.

25. Meeker JR, Strid P, Simeone R, D’Angelo DV, Dieke A, von Essen BS, et al. Pandemic-related stressors and mental health among women with a live birth in 2020. Archives of Women's Mental Health. 2023;26(6):767-76.10.1007/s00737-023-01364-7.
