# Supplementary material for: Associations between food insecurity in high-income countries and pregnancy outcomes: A systematic review and meta-analysis
Source: PLoS Med. 2024 Sep 10;21(9):e1004450. doi: 10.1371/journal.pmed.1004450 (PMC11386426; doi:10.1371/journal.pmed.1004450)
Supplement: S6 Table — (DOCX) [file pmed.1004450.s007.docx]

**Table S6. Newcastle Ottawa scale for quality assessment**

| **Paper** | **Selection*** | | | **Comparability**** | **Outcome**** | | | **Total stars** | **Quality rating** |
| --- | --- | --- | --- | --- | --- | --- | --- | --- | --- |
|  | **Q1** | **Q2** | **Q3** | **Q4** | **Q5** | **Q6** | **Q7** |  |  |
| Richards et al., 2021 [1] | a* | a* | b* | c | a* | a* | a* | 6 | High |
| Luke. 2017 [2] | a* | a* | b* | a* & b* | b* | a* | d | 7 | High |
| Grilo et al., 2015 [3] | b* | a* | b* | a* | b* | b* | d | 4 | Medium |
| Mak. 2019 [4] | a* | a* | b* | b* | c | a* | b* | 6 | High |
| Cheng et al., 2022[5] | b* | a* | b* | b* | b* | a* | b* | 6 | High |
| Mehta et al., 2020 [6] | b* | a* | b* | c | a* | a* | b* | 5 | Medium |
| Eick et al., 2020 [7] | b* | a* | b* | b* | c | a* | a* | 6 | High |
| Laraia et al., 2022 [8] | a* | a* | b* | b* | c | a* | a* | 6 | High |
| Goin et al., 2021 [9] | b* | a* | b* | a* & b* | b* | a* | c | 6 | High |
| Tarasuk et al., 2020 [10] | a* | b* | a* | b* | b* | a* | d | 6 | High |
| Sandoval et al., 2021 [11] | b* | a* | a* | b* | b* | a* | d | 6 | High |
| Sullivan et al., 2021 [12] | a* | a* | b* | c | b* | a* | d | 5 | Medium |
| Power et al., 2017 [13] | a* | a* | b* | b* | b* | a* | a* | 7 | High |
| Tucker et al., 2015 [14] | a* | a* | b* | b* | c | a* | d | 5 | Medium |
| Richards et al., 2020 [15] | a* | a* | b* | b* | c | a* | a* | 6 | High |
| Cooper et al., 2022 [16] | a* | a* | b* | a*& b* | b* | a* | c | 7 | High |
| Tesla et al., 2022 [17] | a* | a* | b* | a*& b* | c | a* | a* | 7 | High |
| Cheu et al., 2020 [18] | b* | a* | b* | a* & b* | b* | a* | b* | 8 | High |
| Bihan et al., 2023 [19] | a* | a* | b* | c | a* | a* | c | 4 | Medium |
| Duh-Leong et al., 2023 [20] | b* | b* | b* | b* | a* | a* | d | 5 | Medium |
| Oresnik., 2020 [21] | b* | b* | b* | b* | a* | a* | d | 6 | High |
| Shriver et al., 2023 [22] | b* | b* | b* | c | a* | a* | c | 5 | Medium |
| Eagleton et al., 2022 [23] | b* | b* | b* | c | a* | a* | b* | 6 | High |
| Joseph et al., 2023 [24] | a* | a* | b* | b* | b* | a* | c | 6 | High |
| Meeker et al., 2023 [25] | a* | a* | b* | b* | c | a* | d | 5 | Medium |
| **Percentage of studies that scored a star for each question:** | **N=25**  **100%** | **N=25**  **100%** | **N=25**  **100%** | **Any star n=19**  **76%**  **(1 star n=14, 2 stars n=5)** | **N=18**  **72%** | **N=25**  **100%** | **N=11**  **44%** | **N=17 High Quality**  **N=8 Medium Quality**  **N=0 Low Quality** | |

*****Q1) Representativeness of the exposed cohort; Q2) Selection of the non-exposed cohort; Q3) Ascertainment of exposure.

**Q4) Comparability of cohorts on the basis of the design or analysis.

***Q5) Assessment of outcome; Q6) Was follow-up long enough for outcomes to occur; Q7) Adequacy of follow up of cohorts.

**References**

1. Richards M, Weigel M, Li M, Rosenberg M, Ludema C. Food insecurity, gestational weight gain and gestational diabetes in the National Children's Study, 2009-2014. J Public Health (Oxf). 2021;43(3):558-66.10.1093/pubmed/fdaa093.

2. Luke S. Neighborhood deprivation, food insecurity and gestational weight gain.: University of South Florida; 2017.

3. Grilo SA, Earnshaw VA, Lewis JB, Stasko EC, Magriples U, Tobin J, et al. Food Matters: Food Insecurity among Pregnant Adolescents and Infant Birth Outcomes. J Appl Res Child. 2015;6(2)

4. Mak J. Food Insecurity During Pregnancy in Canada: University of Toronto; 2019.

5. Cheng ER, Luo M, Perkins M, Blake-Lamb T, Kotelchuck M, Arauz Boudreau A, et al. Household food insecurity is associated with obesogenic health behaviours among a low-income cohort of pregnant women in Boston, MA. Public Health Nutrition. 2022:1-9.10.1017/S1368980022000714.

6. Mehta SS, Applebaum KM, James-Todd T, Coleman-Phox K, Adler N, Laraia B, et al. Associations between sociodemographic characteristics and exposures to PBDEs, OH-PBDEs, PCBs, and PFASs in a diverse, overweight population of pregnant women. J Expo Sci Environ Epidemiol. 2020;30(1):42-55.10.1038/s41370-019-0173-y.

7. Eick SM, Goin DE, Izano MA, Cushing L, DeMicco E, Padula AM, et al. Relationships between psychosocial stressors among pregnant women in San Francisco: A path analysis. PLoS One. 2020;15(6):e0234579.10.1371/journal.pone.0234579.

8. Laraia BA, Gamba R, Saraiva C, Dove MS, Marchi K, Braveman P. Severe maternal hardships are associated with food insecurity among low-income/lower-income women during pregnancy: results from the 2012–2014 California maternal infant health assessment. BMC Pregnancy and Childbirth. 2022;22(1):138.10.1186/s12884-022-04464-x.

9. Goin DE, Izano MA, Eick SM, Padula AM, DeMicco E, Woodruff TJ, et al. Maternal Experience of Multiple Hardships and Fetal Growth: Extending Environmental Mixtures Methodology to Social Exposures. Epidemiology. 2021;32(1):18-26.10.1097/ede.0000000000001272.

10. Tarasuk V, Gundersen C, Wang X, Roth DE, Urquia ML. Maternal Food Insecurity is Positively Associated with Postpartum Mental Disorders in Ontario, Canada. J Nutr. 2020;150(11):3033-40.10.1093/jn/nxaa240.

11. Sandoval VS, Jackson A, Saleeby E, Smith L, Schickedanz A. Associations Between Prenatal Food Insecurity and Prematurity, Pediatric Health Care Utilization, and Postnatal Social Needs. Acad Pediatr. 2021;21(3):455-61.10.1016/j.acap.2020.11.020.

12. Sullivan K, St John M, DeFranco E, Kelly E. Food Insecurity in an Urban Pregnancy Cohort. Am J Perinatol. 2021;40(1):57-61.10.1055/s-0041-1729159.

13. Power M, Uphoff E, Kelly B, Pickett KE. Food insecurity and mental health: an analysis of routine primary care data of pregnant women in the Born in Bradford cohort. J Epidemiol Community Health. 2017;71(4):324-8.10.1136/jech-2016-207799.

14. Tucker CM, Berrien K, Menard MK, Herring AH, Daniels J, Rowley DL, et al. Predicting Preterm Birth Among Women Screened by North Carolina's Pregnancy Medical Home Program. Matern Child Health J. 2015;19(11):2438-52.10.1007/s10995-015-1763-5.

15. Richards M, Weigel M, Li M, Rosenberg M, Ludema C. Household food insecurity and antepartum depression in the National Children's Study. Ann Epidemiol. 2020;44:38-44.e1.10.1016/j.annepidem.2020.01.010.

16. Cooper S, Graham M, Kuo CL, Khangura R, Schmidt A, Bakaysa S. The Relationship between Food Security and Gestational Diabetes among Pregnant Women. AJP Reports. 2022;12(3):E131-E8.doi:10.1055/s-0042-1751082.

17. Testa A, Ganson KT, Jackson DB, Bojorquez-Ramirez P, Weiser SD, Nagata JM. Food insecurity and oral health care experiences during pregnancy: Findings from the Pregnancy Risk Assessment Monitoring System. J Am Dent Assoc. 2022;153(6):503-10.10.1016/j.adaj.2021.12.010.

18. Cheu L, Yee L, Kominiarek M. Food insecurity during pregnancy and gestational weight gain. American journal of obstetrics and gynecology. 2020;220(1):204-.10.1016/j.ajog.2018.11.309.

19. Bihan H, Nachtargeale C, Vicaud E, Sal M, Berkane N, Pinto S, et al. Impact of experiencing multiple vulnerabilities on fetal growth and complications in women with hyperglycemia in pregnancy. BMC Pregnancy Childbirth. 2023;23(1):740.10.1186/s12884-023-06048-9.

20. Duh-Leong C, Perrin EM, Heerman WJ, Schildcrout JS, Wallace S, Mendelsohn AL, et al. Prenatal Risks to Healthy Food Access and High Birthweight Outcomes. Acad Pediatr. 2023.10.1016/j.acap.2023.08.017.

21. Orsenik S. The Intersection of Food Insecurity, Gestational Diabetes and Mental Health Conditions: Examining Pregnancy from a Biocultural Perspective: McCaster University; 2020.

22. Shriver LH, Eagleton SG, Hosseinzadeh M, Buehler C, Wideman L, Leerkes EM. Associations among eating behaviors, food security status, and dietary intake during pregnancy. Appetite. 2023;191:107062.<https://doi.org/10.1016/j.appet.2023.107062>.

23. Eagleton SG, Shriver LH, Buehler C, Wideman L, Leerkes EM. Longitudinal Associations Among Food Insecurity During Pregnancy, Parental Mental Health Symptoms, Controlling Feeding Styles, and Infant Food Responsiveness. The Journal of Nutrition. 2022;152(12):2659-68.10.1093/jn/nxac225.

24. Joseph NT, Stanhope KK, Geary F, McIntosh M, Platner MH, Wichmann HK, et al. Social Determinants of Health Needs and Perinatal Risk in Socially Vulnerable Pregnant Patients. J Health Care Poor Underserved. 2023;34(2):685-702.10.1353/hpu.2023.0058.

25. Meeker JR, Strid P, Simeone R, D’Angelo DV, Dieke A, von Essen BS, et al. Pandemic-related stressors and mental health among women with a live birth in 2020. Archives of Women's Mental Health. 2023;26(6):767-76.10.1007/s00737-023-01364-7.
