# Supplementary material for: Associations between food insecurity in high-income countries and pregnancy outcomes: A systematic review and meta-analysis
Source: PLoS Med. 2024 Sep 10;21(9):e1004450. doi: 10.1371/journal.pmed.1004450 (PMC11386426; doi:10.1371/journal.pmed.1004450)
Supplement: S7 Table — (DOCX) [file pmed.1004450.s008.docx]

**Table S7. Association between food insecurity and maternal physical health in pregnancy outcomes**

| **Study** | **Outcome definition** | **Measurement of FI** | **Reference** | **Level of FI** | **Sample size** | **Results** | **Adjustments** |
| --- | --- | --- | --- | --- | --- | --- | --- |
| **Gestational diabetes mellitus (GDM)** | | | | | | | |
| Sullivan et al., 2021 [1] | GDM | USDA HFSSM 3 items | FS | FI | 426 | COR 1.02 (95% CI 0.40, 2.57)* | None |
| Richards et al., 2021 [2] | GDM | USDA HFSSM 6 items | FS | Marginally FS and FI | 592 | **OR 1.97 (95% CI 1.01, 3.84)** | None |
|  | GDM |  | FS | Marginally FS and FI | 592 | AOR 1.12 (95% CI 0.47, 2.66)* | Age, race, medical insurance, income, pre-pregnancy BMI and site. |
|  | GDM |  | FS | Marginally FS | 523 | AOR 0.69 (95% CI 0.19, 2.45) |  |
|  | GDM |  | FS | FI | 542 | AOR 1.02 (95% CI 0.38, 2.76) |  |
| Luke et al., 2017 [3] | GDM | 1 question rapid assessment tool | FS | FI | 9547 | **COR 1.65 (95% CI 1.35, 2.02)*** | None |
| Cheng et al., 2022 [4] | GDM | Hunger vital sign 2 screening items | FS | FI | 858 | AOR 1.60 (95% CI 0.79, 3.26)* | Maternal race/ethnicity, annual household income, marital status and education. |
| Cooper et al., 2022 [5] | GDM | USDA HFSSM 18 items | FS | FI | 70 | **OR 5.02 (95% CI 1.77, 15.52)** | NR |
|  | GDM |  | FS | FI | 70 | **AOR 7.05 (95% CI 1.78, 34.72) *** | Age, race, and pre-pregnancy BMI. |
| Cheu et al., 2020 [6] | GDM | USDA HFSSM 10 items | Adequate FS | Inadequate FS | 299 | COR 1.80 (95% CI 0.48, 6.62)* | None |
| Joseph et al., 2023 [7] | GDM | Hunger vital sign 2 screening items | Not FI | FI | 1,065 | ARR 1.16 (95%CI 0.76, 1.77) | Maternal age at delivery, insurance type, and parity |
| Shriver et al., 2023 [8] | GDM | USDA HFSSM 6 items | FS | FI | 297 | COR 0.96 (95%CI 0.37, 2.47)*. 2020 | None |
| Oresnik. 2020 [9] | GDM | USDA HFSSM 18 items | FS | FI | 3,262 | **AOR 5.107 (95%CI 1.94, 13.46)*** | Maternal age, income, and number of dependents |
| Oresnik. 2020 [9] | GDM | 1 question adapted from USDA HFSSM | FS | FI | 330 | AOR 0.261 (95%CI 0.035, 1.929)* | Maternal age, number of dependents, ethnicity, and pre-pregnancy BMI |
| **Outcomes related to GDM** | | | | | | | |
| Cheng et al., 2022 [4] | Isolated hyperglycemia | Hunger vital sign 2 screening items | FS | FI | 710 | AOR 0.89 (95% CI 0.42, 1.89) | Maternal race/ethnicity, annual household income, marital status and education. |
| Cheng et al., 2022 [4] | Impaired glucose tolerance |  | FS | FI | 710 | AOR 1.04 (95% CI 0.36, 3.02) |  |
| **Hypertensive disorders** | | | | | | | |
| Sullivan et al., 2021 [1] | Pre-eclampsia | USDA HFSSM 3 items | FS | FI | 426 | **COR 1.91 (95% CI 1.11, 3.29)** | None |
|  | Chronic hypertension |  | FS | FI | 426 | COR 1.45 (95% CI 0.65, 3.24) | None |
| Luke et al., 2017 [3] | Gestational hypertension | 1 question rapid assessment tool | FS | FI | 9555 | COR 1.22 (95% CI 0.94, 1.59) | None |
| Tarasuk et al., 2020 [10] | Hypertension or diabetes | USDA HFSSM 18 items | FS | Marginal FI | 1790 | ARR 0.99 (95% CI 0.51, 1.92) | Age, partnership status, previous live birth, housing tenure, rural residence, household income, immigration status, race, education. |
|  |  |  |  | Moderate/ severe FI | 1876 | ARR 1.44 (95% CI 0.88, 2.35) |  |
| Bihan et al. 2023 [11] | Hypertensive disorders | Single item | Not FI | FI | 887 | COR 16.94 (95%CI 7.79, 36.85) | None |
|  | Pre-eclampsia | Single item | Not FI | FI | 887 | COR 1.78 (95%CI 0.77, 4.10) | None |
| Joseph et al., 2023 [7] | Preeclampsia without severe features or gestational hypertension | Hunger vital sign 2 screening items | Not FI | FI | 1,065 | ARR 0.91 (95%CI 0.8, 1.03) | Maternal age at delivery, insurance type, and parity |
|  | Preeclampsia with severe features | Hunger vital sign 2 screening items | Not FI | FI | 1,065 | ARR 0.95 (95%CI 0.78, 1.16) | Maternal age at delivery, insurance type, and parity |
| **Mode of delivery** | | | | | | | |
| Cooper et al., 2022 [5] | Caesarean | USDA HFSSM 18 items | FS | FI | 70 | OR 2.59 (95% CI 0.94, 7.16)* | None |
| Cheu et al., 2020 [6] | Caesarean | USDA HFSSM 10 items | Adequate FS | Inadequate FS | 299 | COR 1.75 (95% CI 0.82, 3.72)* | None |
|  | Vacuum or forceps-assisted |  | Adequate FS | Inadequate FS | 299 | COR 0.96 (95% CI 0.11, 7.98) | None |
| Bihan et al. 2023 [11] | Caesarean | Single item | Not FI | FI | 887 | COR 0.94 (95%CI 0.63, 1.38)* | None |
| Joseph et al., 2023 [7] | Caesarean | Hunger vital sign 2 screening items | Not FI | FI | 1,065 | ARR 0.84 (95%CI 0.61, 1.16) | Maternal age at delivery, insurance type, and parity |
| **Oral health** | | | | | | | |
| Testa et al., 2022 [12] | Need to see a dentist for a problem | 1 item from USDA HFSSM | FI | FS | 21080 | **AOR 1.91 (95% CI 1.62, 2.25)** | Maternal age, race/ethnicity, education, marital status, number of prior births, pre-pregnancy BMI, household income, number of financial dependents, dental insurance status. |
|  | Went to see a dentist for a problem |  | FI | FS | 21080 | **AOR 1.42 (95% CI 1.18, 1.71)** |  |
|  | Did not know it was important to care for teeth |  | FI | FS | 21080 | **AOR 1.29 (95% CI 1.04, 1.59)** |  |
|  | Did not talk about dental health with an oral health provider |  | FI | FS | 21080 | **AOR 1.54 (95% CI 1.32, 1.80)** |  |
|  | Did not receive dental prophylaxis during pregnancy |  | FI | FS | 21080 | **AOR 1.38 (95% CI 1.17, 1.64)** |  |
|  | Saw dentist conditional of having a problem |  | FI | FS | 21080 | AOR 0.66 (95% CI 0.50, 0.86) |  |
| **Serum concentration of organohalogens chemicals** | | | | | | | |
| Mehta et al., 2020 [13] | In-PFNA | USDA HFSSM 10 items | Marginal/High FS | Low/Very Low FS | 98 | β 0.04 (95% CI −0.18, 0.25) | None |
|  | ln-PFDeA |  | Marginal/High FS | Low/Very Low FS | 98 | β −0.07 (95% CI −0.41, 0.28) |  |
|  | ln-PFOS |  | Marginal/High FS | Low/Very Low FS | 98 | **β 0.27 (95% CI 0.04, 0.50)** |  |
|  | ln-PFOA |  | Marginal/High FS | Low/Very Low FS | 98 | β 0.13 (95% CI −0.19, 0.45) |  |
|  | ln-PFHxS |  | Marginal/High FS | Low/Very Low FS | 98 | β 0.14 (95% CI −0.20, 0.47) |  |
|  | ln-ΣPFAS |  | Marginal/High FS | Low/Very Low FS | 98 | β 0.19 (95% CI −0.003, 0.38) |  |
| **Other outcomes** | | | | | | | |
| Cheu et al., 2020 [6] | Postpartum haemorrhage | USDA HFSSM 10 items | Adequate FS | Inadequate FS | 299 | COR 1.80 (95% CI 0.48, 6.62) | None |
| Tarasuk et al., 2020 [10] | Admission to hospital during pregnancy | USDA HFSSM 18 items | FS | Marginal FI | 1790 | ARR 1.09 (95% CI 0.57, 2.09) | Age, partnership status, previous live birth, housing tenure, rural residence, main household income, immigration status, race, education. |
|  |  |  |  | Moderate/severe FI | 1876 | ARR 0.94 (95% CI 0.55, 1.60) |  |
|  | Length of stay in hospital for childbirth episode, day |  |  | Marginal FI | 1790 | Mean difference 1.00 (95% CI 0.89, 1.11) |  |
|  |  |  |  | Moderate/severe FI | 1876 | Mean difference 1.03 (95% CI 0.94, 1.13) |  |
| Joseph et al., 2023 [7] | Severe Maternal Morbidity | Hunger vital sign 2 screening items | Not FI | FI | 1,065 | ARR 1.05 (95%CI 0.69, 1.6) | Maternal age at delivery, insurance type, and parity |
|  | Non-Transfusion Severe Maternal Morbidity |  | Not FI | FI | 1,065 | ARR 1.12 (95%CI 0.60, 2.12) | Maternal age at delivery, insurance type, and parity |

**Bold** indicates statistically significant result. *Results included in meta-analysis. FI - Food Insecurity; FS - Food Security; CI - Confidence Interval; OR – Odds Ratio; AOR - Adjusted Odds Ratio; COR – Calculated OR; RR- Relative Risk ; ARR – Adjusted RR; In – log transformed; PFNA – perfluorononanoic acid; PFDeA - perfluorodecanoic acid; PFOS - perfluorooctane sulfonate; PFOA - perfluorooctanoic acid; PFHxS - perfluorohexane sulfonate; ΣPFAS – sum of perfluoroalkyl substances

**References**

1. Sullivan K, St John M, DeFranco E, Kelly E. Food Insecurity in an Urban Pregnancy Cohort. Am J Perinatol. 2021;40(1):57-61.10.1055/s-0041-1729159.

2. Richards M, Weigel M, Li M, Rosenberg M, Ludema C. Food insecurity, gestational weight gain and gestational diabetes in the National Children's Study, 2009-2014. J Public Health (Oxf). 2021;43(3):558-66.10.1093/pubmed/fdaa093.

3. Luke S. Neighborhood deprivation, food insecurity and gestational weight gain.: University of South Florida; 2017.

4. Cheng ER, Luo M, Perkins M, Blake-Lamb T, Kotelchuck M, Arauz Boudreau A, et al. Household food insecurity is associated with obesogenic health behaviours among a low-income cohort of pregnant women in Boston, MA. Public Health Nutrition. 2022:1-9.10.1017/S1368980022000714.

5. Cooper S, Graham M, Kuo CL, Khangura R, Schmidt A, Bakaysa S. The Relationship between Food Security and Gestational Diabetes among Pregnant Women. AJP Reports. 2022;12(3):E131-E8.doi:10.1055/s-0042-1751082.

6. Cheu L, Yee L, Kominiarek M. Food insecurity during pregnancy and gestational weight gain. American journal of obstetrics and gynecology. 2020;220(1):204-.10.1016/j.ajog.2018.11.309.

7. Joseph NT, Stanhope KK, Geary F, McIntosh M, Platner MH, Wichmann HK, et al. Social Determinants of Health Needs and Perinatal Risk in Socially Vulnerable Pregnant Patients. J Health Care Poor Underserved. 2023;34(2):685-702.10.1353/hpu.2023.0058.

8. Shriver LH, Eagleton SG, Hosseinzadeh M, Buehler C, Wideman L, Leerkes EM. Associations among eating behaviors, food security status, and dietary intake during pregnancy. Appetite. 2023;191:107062.<https://doi.org/10.1016/j.appet.2023.107062>.

9. Orsenik S. The Intersection of Food Insecurity, Gestational Diabetes and Mental Health Conditions: Examining Pregnancy from a Biocultural Perspective: McCaster University; 2020.

10. Tarasuk V, Gundersen C, Wang X, Roth DE, Urquia ML. Maternal Food Insecurity is Positively Associated with Postpartum Mental Disorders in Ontario, Canada. J Nutr. 2020;150(11):3033-40.10.1093/jn/nxaa240.

11. Bihan H, Nachtargeale C, Vicaud E, Sal M, Berkane N, Pinto S, et al. Impact of experiencing multiple vulnerabilities on fetal growth and complications in women with hyperglycemia in pregnancy. BMC Pregnancy Childbirth. 2023;23(1):740.10.1186/s12884-023-06048-9.

12. Testa A, Ganson KT, Jackson DB, Bojorquez-Ramirez P, Weiser SD, Nagata JM. Food insecurity and oral health care experiences during pregnancy: Findings from the Pregnancy Risk Assessment Monitoring System. J Am Dent Assoc. 2022;153(6):503-10.10.1016/j.adaj.2021.12.010.

13. Mehta SS, Applebaum KM, James-Todd T, Coleman-Phox K, Adler N, Laraia B, et al. Associations between sociodemographic characteristics and exposures to PBDEs, OH-PBDEs, PCBs, and PFASs in a diverse, overweight population of pregnant women. J Expo Sci Environ Epidemiol. 2020;30(1):42-55.10.1038/s41370-019-0173-y.
