# Supplementary material for: Associations between food insecurity in high-income countries and pregnancy outcomes: A systematic review and meta-analysis
Source: PLoS Med. 2024 Sep 10;21(9):e1004450. doi: 10.1371/journal.pmed.1004450 (PMC11386426; doi:10.1371/journal.pmed.1004450)
Supplement: S8 Table — (DOCX) [file pmed.1004450.s009.docx]

**Table S8. Association between food insecurity and maternal mental health outcomes**

| **Study** | **Outcome definition** | **Measurement of FI** | **Reference** | **Level of FI** | **Sample size** | **Results** | **Adjustments** |
| --- | --- | --- | --- | --- | --- | --- | --- |
| **Depression** | | | | | | | |
| Cheng et al., 2022 [1] | Current depression | Hunger vital sign 2 screening items | FS | FI | 720 | **AOR 4.42 (95% CI 2.33, 8.35)** | Maternal race/ethnicity, annual household income, marital status and education. |
| Eick et al., 2020 [2] | Depression (continuous) | 1 screening question | FS | FI | 510 | **β 2.67 (95% CI 1.31, 4.04)** | Maternal age, race, education. |
| Richards et al., 2020 [3] | Antepartum depression CES-D score >21 | USDA HFSSM 6 items | FS | FI | 752 | **Weighted AOR 3.68 (95% CI 1.43, 9.43)** | Age, race, income, marital status, health insurance, social support, adults in households, history of depression, history of anxiety, previous lost pregnancy, pregnancy intention, site. |
|  |  |  | FS | Marginally FS | 752 | Weighted AOR 2.97 (95% CI 0.85, 10.35) |  |
|  | Antepartum depression CES-D score >24 |  | FS | FI | 752 | **Weighted AOR 4.01 (95% CI 1.42, 11.90)** |  |
|  |  |  | FS | Marginally FS | 752 | Weighted AOR 3.64 (95% CI 0.77, 17.31) |  |
| **Depressive symptoms** | | | | | | | |
| Laraia et al., 2022 [4] | Prenatal depressive symptoms | USDA HFSSM 6 items | FS | Marginally FS | 14274 | **ARRR 1.75 (95% CI 1.36, 2.23)** | Year of survey, maternal race/ethnicity, age, education, language spoken at home, number in the household, income as the percent of the poverty guidelines, insurance coverage, marital status |
|  |  |  | FS | Low FS | 14274 | **ARRR 1.72 (95% CI 1.40, 2.11)** |  |
|  |  |  | FS | Very low FS | 14274 | **ARRR 3.26 (95% CI 2.46, 4.32)** |  |
| Girlo et al., 2015 [5] | Depressive symptom scores (continuous) | 1 screening question | FS: Mean 9.30 (SD 7.40) | Acute FI | 647 | **FI: Mean 11.10 (SD 7.80) p<0.001** | None |
|  |  |  |  | Chronic FI | 651 | **FI: Mean 13.60 (SD 8.80) p<0.001** |  |
| Eagleton et al., 2022 [6] | Depressive symptom scores (continuous) | USDA HFSSM 6 items | **FS: Mean 0.49 (0.32)** | **FI** | **168** | **FI: Mean 0.81 (0.46), p<0.001** | None |
| Meeker et al. 2023 [7] | Feeling more depressed than usual | 1 question | Not FI | FI | 15525 | **APR 2.32 (95%CI 2.13–2.53)** | Maternal age, race/ethnicity, education level, health insurance at delivery, and jurisdiction of residence |
| **Stress** | | | | | | | |
| Cheng et al., 2022 [1] | High stress | Hunger vital sign 2 screening items | FS | FI | 856 | **AOR 2.91 (95% CI 1.98, 4.28)*** | Maternal race/ethnicity, annual household income, marital status and education. |
| Luke. 2017 [8] | Stress (1-2 events) | 1 question rapid assessment tool | FS | FI | 7279 | **OR 3.11 (95% CI 2.21, 4.37)** | None |
|  | Stress (3-5 events) |  | FS | FI | 4992 | **OR 12.46 (95% CI 8.99, 17.27)** | None |
|  | Stress (6 or more events) |  | FS | FI | 3257 | **OR 46.24 (95% CI 32.77, 65.26)** | None |
|  |  |  | FS | FI | 3257 | **AOR 25.00 (95% CI 13.60, 45.70)*** | Age, previous live birth, education, BMI, race, WIC use. |
| Mak. 2019 [9] | High levels of stress | USDA HFSSM 10 items | FS | Marginally FI | 4754 | AOR 1.41 (95% CI 0.86, 2.30)* | Household income, age, post-secondary education, indigeneity, two-year cycle. |
|  |  |  |  | Moderate/ severe FI | 4754 | **AOR 2.82 (95% CI 1.90, 4.18)*** |  |
| Eick et al., 2020 [2] | Perceived Stress | 1 screening question | FS | FI | 510 | β 0.52 (95% CI -0.17, 1.33) | Maternal age, race, education. |
| **Anxiety** | | | | | | | |
| Cheng et al., 2022 [1] | Anxiety score (continuous) | Hunger vital sign 2 screening items | FS | FI | 858 | **Mean difference 1.55 (95% CI 1.04, 2.05)** | Maternal race/ethnicity, annual household income, marital status and education. |
| Mak. 2019 [9] | Diagnosed anxiety disorder | USDA HFSSM 10 items | FS | Marginally FI | 4754 | AOR 1.82 (95% CI 0.94, 3.54) | Household income, age, post-secondary education, indigeneity, two-year cycle. |
|  |  |  |  | Moderate/ severe FI | 4754 | **AOR 3.23 (95% CI 1.92, 5.43)** |  |
| Girlo et al., 2015 [5] | Generalized anxiety score (continuous) | 1 screening question | **FS: Mean 4.60 (SD 4.50)** | Acute FI | 647 | **FI: Mean 6.00 (SD 5.50), p<0.001** | None |
|  |  |  |  | Chronic FI | 651 | **FI: Mean 6.60 (SD 5.30) p<0.001** |  |
| Meeker et al. 2023 [7] | Feeling more anxious than usual | 1 question | Not FI | FI | 15525 | **APR 1.79 (95%CI 1.71–1.88)** | Maternal age, race/ethnicity, education level, health insurance at delivery, and jurisdiction of residence |
| Eagleton et al., 2022 [6] | Anxiety symptom scores | USDA HFSSM 6 items | **FS: Mean 1.71 (0.32)** | **FI** | **168** | **FI: Mean 2.04 (0.51), p<0.001** | None |
| Orsenik. 2020 [10] | Anxiety disorder | USDA HFSSM 18 items | FS | FI | 3,262 | **AOR 2.49 (95%CI 1.09, 5.67)** | Having a mood disorder, level of education, and number of dependents |
| **Other disorder** | | | | | | | |
| Mak. 2019 [9] * | Diagnosed mood disorder | USDA HFSSM 10 items | FS | Marginally FI | 4754 | **AOR 2.29 (95% CI 1.09, 4.80)** | Household income, age, post-secondary education, indigeneity, two-year cycle. |
|  |  |  |  | Moderate/ severe FI | 4754 | **AOR 4.82 (95% CI 2.80, 8.28)** |  |
| Cheu et al., 2020 [11]* | Mood disorder | USDA HFSSM 10 items | Adequate FS | Inadequate FS | 299 | COR 1.63 (95% CI 0.76, 3.49) | None |
| Power et al., 2017 [12] | Common mental disorders | USDA HFSSM 18 items | FS | FI | 1280 | **IRR 1.90 (95% CI 1.30, 2.80)**** | None |
| Orsenik. 2020 [10] | Mood disorder | USDA HFSSM 18 items | FS | FI | 3,262 | AOR 1.75 (95% CI 0.67, 4.56) | Having a mood disorder, level of education, and number of dependents |
| **Other outcomes relating to mental health** | | | | | | | |
| Sullivan et al., 2021 [13] | Measures of resilience:  Feel little to no love in life | USDA HFSSM 3 items | FS | FI | 426 | **COR 9.33 (95% CI 3.39, 25.69)** | None |
|  | Feel dissatisfied or very dissatisfied with life |  | FS | FI | 426 | **COR 7.22 (95% CI 3.11 to 16.74)** |  |
|  | Feel little to no joy in the past year |  | FS | FI | 426 | **COR 6.79 (95% CI 3.36 to 13.73)** |  |
|  | Felt a moderate to extreme amount of despair in the past year |  | FS | FI | 426 | **COR 2.24 (95% CI 1.38 to 3.65)** |  |
|  | Feel moderate or less control over own life |  | FS | FI | 426 | **COR 3.69 (95% CI 1.86 to 7.33)** |  |
|  | Experienced physical abuse during pregnancy |  | FS | FI | 426 | COR 0.58 (95% CI 0.07 to 4.75) |  |
| Mak. 2019 [9] | Poor/fair perceived health | USDA HFSSM 10 items | FS | Marginally FI | NR | AOR 1.60 (95% CI 0.60, 4.26) | Household income, age, post-secondary education, indigeneity, two-year cycle. |
|  |  |  | FS | Moderate/ severe FI | NR | AOR 1.52 (95% CI 0.82, 2.80) |  |
|  | Poor/fair perceived mental health |  | FS | Marginally FI | NR | AOR 2.23 (95% CI 0.64, 7.78) |  |
|  |  |  | FS | Moderate/ severe FI | NR | **AOR 3.79 (95% CI 1.52, 9.48)** |  |
|  | Worse health compared to 1 year prior |  | FS | Marginally FI | NR | AOR 0.96 (95% CI 0.38, 2.39) |  |
|  |  |  | FS | Moderate/ severe FI | NR | AOR 0.91 (95% CI 0.54, 1.53) |  |
|  | Weak sense of community |  | FS | Marginally FI | NR | AOR 1.08 (95% CI 0.72, 1.62) |  |
|  |  |  | FS | Moderate/ severe FI | NR | AOR 1.33 (95% CI 0.92, 1.92) |  |
| Eick et al., 2020 [2] | Community Status | 1 screening question | FS | FI | 510 | β -0.02 (95% CI -0.10, 0.02) | Maternal age, race, education. |
| Eagleton et al., 2022 [6] | Parental mental health symptoms | USDA HFSSM 6 items | **FS** | **FI** | 168 | β 0.54, p<0.001 | Birth parent age, race and ethnicity, educational attainment, income-to-needs ratio, residing with an intimate partner, WIC participation, and breastfeeding intensity |

**Bold** indicates statistically significant result. * Results included in meta-analysis. ** Combined pre-birth and pregnancy data periods due to inability to access individual data time points from author. FI - Food Insecurity; FS - Food Security; NR - Not Reported; CI - Confidence Interval; OR – Odds Ratio; AOR - Adjusted Odds Ratio; COR- calculated Odds Ratio; ARRR- Adjusted Relative Risk Ratio; IRR-Incidence Rate Ratio; SD – Standard Deviation; CES-D- Center for Epidemiologic Studies Depression Scale; APR- adjusted prevalence ratio

**References**

1. Cheng ER, Luo M, Perkins M, Blake-Lamb T, Kotelchuck M, Arauz Boudreau A, et al. Household food insecurity is associated with obesogenic health behaviours among a low-income cohort of pregnant women in Boston, MA. Public Health Nutrition. 2022:1-9.10.1017/S1368980022000714.

2. Eick SM, Goin DE, Izano MA, Cushing L, DeMicco E, Padula AM, et al. Relationships between psychosocial stressors among pregnant women in San Francisco: A path analysis. PLoS One. 2020;15(6):e0234579.10.1371/journal.pone.0234579.

3. Richards M, Weigel M, Li M, Rosenberg M, Ludema C. Household food insecurity and antepartum depression in the National Children's Study. Ann Epidemiol. 2020;44:38-44.e1.10.1016/j.annepidem.2020.01.010.

4. Laraia BA, Gamba R, Saraiva C, Dove MS, Marchi K, Braveman P. Severe maternal hardships are associated with food insecurity among low-income/lower-income women during pregnancy: results from the 2012–2014 California maternal infant health assessment. BMC Pregnancy and Childbirth. 2022;22(1):138.10.1186/s12884-022-04464-x.

5. Grilo SA, Earnshaw VA, Lewis JB, Stasko EC, Magriples U, Tobin J, et al. Food Matters: Food Insecurity among Pregnant Adolescents and Infant Birth Outcomes. J Appl Res Child. 2015;6(2)

6. Eagleton SG, Shriver LH, Buehler C, Wideman L, Leerkes EM. Longitudinal Associations Among Food Insecurity During Pregnancy, Parental Mental Health Symptoms, Controlling Feeding Styles, and Infant Food Responsiveness. The Journal of Nutrition. 2022;152(12):2659-68.10.1093/jn/nxac225.

7. Meeker JR, Strid P, Simeone R, D’Angelo DV, Dieke A, von Essen BS, et al. Pandemic-related stressors and mental health among women with a live birth in 2020. Archives of Women's Mental Health. 2023;26(6):767-76.10.1007/s00737-023-01364-7.

8. Luke S. Neighborhood deprivation, food insecurity and gestational weight gain.: University of South Florida; 2017.

9. Mak J. Food Insecurity During Pregnancy in Canada: University of Toronto; 2019.

10. Orsenik S. The Intersection of Food Insecurity, Gestational Diabetes and Mental Health Conditions: Examining Pregnancy from a Biocultural Perspective: McCaster University; 2020.

11. Cheu L, Yee L, Kominiarek M. Food insecurity during pregnancy and gestational weight gain. American journal of obstetrics and gynecology. 2020;220(1):204-.10.1016/j.ajog.2018.11.309.

12. Power M, Uphoff E, Kelly B, Pickett KE. Food insecurity and mental health: an analysis of routine primary care data of pregnant women in the Born in Bradford cohort. J Epidemiol Community Health. 2017;71(4):324-8.10.1136/jech-2016-207799.

13. Sullivan K, St John M, DeFranco E, Kelly E. Food Insecurity in an Urban Pregnancy Cohort. Am J Perinatol. 2021;40(1):57-61.10.1055/s-0041-1729159.
