# Supplementary material for: Associations between food insecurity in high-income countries and pregnancy outcomes: A systematic review and meta-analysis
Source: PLoS Med. 2024 Sep 10;21(9):e1004450. doi: 10.1371/journal.pmed.1004450 (PMC11386426; doi:10.1371/journal.pmed.1004450)
Supplement: S9 Table — (DOCX) [file pmed.1004450.s010.docx]

**Table S9. Association between food insecurity and infant health outcomes**

| **Study** | **Outcome definition** | **Measurement of FI** | **Reference category** | **Level of FI** | **Sample size** | **Results** | **Adjustments** |
| --- | --- | --- | --- | --- | --- | --- | --- |
| **Birthweight outcomes** | | | | | | | |
| Cheu et al., 2020 [1] | Birthweight | USDA HFSSM 10 items | Median 3267.00g (IQR 2980.00, 3600.00g) | Inadequate FS | 299 | FI: Median 3185g (IQR 2810.00, 3550.00g) p=0.28 | None |
| Grilo et al., 2015 [2] | Birthweight | 1 screening question | FS: Mean 3213.60g (SD 527.90g) | Acute FI | 647 | FI: Mean 3197.00g (SD 447.00g) p=0.57 | None |
|  |  |  | FS: Mean 3197.1g (SD 527.9g) | Chronic FI | 651 | FI: Mean 3167.20g (SD 591.10g) p=0.57 | None |
| Goin et al., 2021[3] | Birthweight | USDA HFSSM | FS: Mean 3363.0g (SD 574.0g) | FI | 510 | FI: Mean 3170.00g (SD 628.00g) p: NR | None |
|  | Difference in birthweight (individual) |  | FS | FI | 510 | MD -139.80g (95% CI -306.70, 27.20) | Maternal age, educational attainment, race/ethnicity, marital status, parity, and pre-pregnancy BMI. |
|  | Difference in birthweight (mutually adjusted) |  | FS | FI | 510 | MD -154.10g (95% CI -333.50, 25.30) |  |
|  | Difference in birthweight by gestational age z-score (individual) |  | FS | FI | 510 | MD -0.16 (95% CI -0.45, 0.14) |  |
|  | Difference in birthweight by gestational age z-score (mutually adjusted) |  | FS | FI | 510 | MD -0.21 (95% CI -0.53, 0.11) |  |
|  | Birthweight for gestational age z-score |  | FS: Mean 0.10g (SD 1.07g) | FI | 510 | FI: Mean -0.10g (SD 1.04g) p: NR | None |
| Eagleton et al. 2022 [4] | Birthweight | USDA HFSSM 6 items | FS: Mean 3.35g (0.49) | FI | 168 | FI: Mean 3.34g (0.46), p=0.954 | None |
| **Low birthweight and small for gestational age (SGA)** | | | | | | | |
| Sullivan et al., 2021 [5] | Low birthweight (not defined) | USDA HFSSM 3 items | FS | FI | 426 | RR 1.21 (p=0.51) | NR |
| Cheu et al., 2020 [1] | Low birthweight (birthweight <2,500g) | USDA HFSSM 10 items | Adequate FS | Inadequate FS | 299 | COR 1.39 (95% CI 0.45, 4.29) | None |
|  | SGA (birthweight <10th percentile) |  | Adequate FS | Inadequate FS | 299 | COR 0.79 (95% CI (0.17, 3.55)* | None |
| Luke. 2017 [6] | SGA (birthweight <10th percentile) | 1 question rapid assessment tool | FS | FI | 9555 | **COR 1.45 (95% CI 1.22, 1.72)*** | None |
| Tarasuk et al., 2020 [7] | SGA (Sex- and gestational age-specific birth weight below the 10th percentile based on a Canadian reference) | USDA HFSSM 18 items | FS | FI (combined marginal, moderate/ severe) | 1998 | COR 0.85 (95% CI 0.53, 1.38)* | None |
|  |  |  | FS | Marginal FI | 1790 | ARR 0.57 (95% CI 0.26, 1.27) | Age, partnership status, previous live birth, housing tenure, rural residence, main income source of the household, immigration status, race and education level. |
|  |  |  | FS | Moderate/ severe FI | 1876 | ARR 0.80 (95% CI 0.47, 1.37) |  |
| Bihan et al. 2023 [8] | SGA | Single item | Not FI | FI | 887 | COR 1.23 (95%CI 0.73, 2.06)* | None |
| **Large for gestational age (LGA)** | | | | | | | |
| Luke. 2017 [6] | LGA (birthweight >90th percentile) | 1 question rapid assessment tool | FS | FI | 9555 | COR 0.75 (95% CI 0.55, 1.02)* | None |
| Cheu et al., 2020 [1] | LGA (birthweight >90th percentile) | USDA HFSSM 10 items | Adequate FS | Inadequate FS | 299 | COR 1.19 (95% CI (0.42, 3.39)* | None |
| Tarasuk et al., 2020 [7] | LGA (Sex- and gestational age-specific birthweight >the 90th percentile based on a Canadian reference) | USDA HFSSM 18 items | FS | FI (combined marginal, moderate/ severe) | 1998 | COR 1.06 (95% CI 0.74, 1.53)* | None |
|  |  |  | FS | Marginal FI | 1790 | ARR 1.13 (95% CI 0.68, 1.87) | Age, partnership status, previous live birth, housing tenure, rural residence, main income source of the household, immigration status, race and education level. |
|  |  |  | FS | Moderate/ severe FI | 1876 | ARR 1.14 (95% CI 0.75, 1.72) |  |
| Bihan et al. 2023 [8] | LGA | Single item | Not FI | FI | 887 | COR 0.87 (95%CI 0.50, 1.49)* | None |
| Duh-Leong et al. 2023 [9] | High birthweight^ | USDA HFSSM 5 items | Not FI | FI | 787 | **AOR 2.07 (95%CI 1.07, 3.98)** | Child sex, race and ethnicity, birthing parent age, education, income, and Greenlight Plus study site |
| Duh-Leong et al. 2023[9] | High birthweight^ | USDA HFSSM 5 items | Not FI | FI | 787 | **AOR 1.96 (95%CI 1.01, 3.82)** | Poor neighbourhood food environment, child sex, race and ethnicity, birthing parent age, education, income, and Greenlight Plus study site |
| **Preterm delivery** | | | | | | | |
| Sullivan et al., 2021 [5] | Pre-term birth (not defined) | USDA HFSSM 3 items | FS | FI | 426 | RR 1.27 (p=0.45) | None |
| Tucker et al., 2015[10] | 24-36 weeks | 1 screening question | FS | FI | 15428 | OR 1.16 (95% CI 0.93, 1.43)* | None |
|  | Pre-term birth and parity |  | FS | FI | 15428 | **OR 1.41 (95% CI 1.04, 1.91)** | None |
| Sandoval et al., 2021 [11] | <37 weeks | USDA HFSSM 6 items | FS | FI | 268 | **AOR 3.00 (95% CI 1.00, 8.90)*** | Mother’s race, ethnicity, preferred language, education level, employment status, number of prior gestations, number of prior live births, relationships with the child's father, and the presence of a partner in the home. |
|  | <36 weeks |  |  |  |  | **AOR 4.80 (95% CI 1.40, 16.30)** |  |
|  | <35 weeks |  |  |  |  | **AOR 5.30 (95% CI 1.10, 26.20)** |  |
|  | <34 weeks |  |  |  |  | **AOR 8.70 (95% CI 1.00, 79.10)** |  |
| Tarasuk et al., 2020 [7] | <37 weeks | USDA HFSSM 18 items | FS | FI (combined marginal, moderate/ severe) | 1998 | COR 1.10 (95% CI 0.65, 1.85)* | None |
| Cheu et al., 2020 [1] | <37 weeks | USDA HFSSM 10 items | Adequate FS | Inadequate FS | 299 | COR 1.96 (95% CI 0.74, 5.18)* | None |
| Joseph et al., 2023 [12] | Preterm birth (< 37 weeks) | Hunger vital sign 2 screening items | Not FI | FI | 1,065 | ARR 1.04 (95%CI 0.72, 1.51) | Maternal age at delivery, insurance type, and parity |
| Bihan et al. 2023 [8] | Preterm birth (< 37 weeks) | Single item | Not FI | FI | 887 | COR 0.79 (95%CI 0.38, 1.65)* | None |
| **Admission to NICU** | | | | | | | |
| Cooper et al., 2022 [13] | Admission to NICU |  | FS | FI | 70 | **COR 4.77 (95% CI 1.07, 21.22)*** | None |
| Cheu et al., 2020 [1] | Admission to NICU | USDA HFSSM 10 items | Adequate FS | Inadequate FS | 299 | **COR 2.78 (95% CI 1.22, 6.32)*** | None |
| Tarasuk et al., 2020 [7] | Admission to NICU | USDA HFSSM 18 items | FS | FI (combined marginal, moderate/ severe) | 1998 | COR 1.08 (95% CI 0.74, 1.56)* | None |
|  | Admission to NICU |  | FS | Marginal FI | 1790 | ARR 0.75 (95% CI 0.41, 1.38) | Age, partnership status, previous live birth, housing tenure, rural residence, main income source of the household, immigration status, race and education level. |
|  | Admission to NICU |  | FS | Moderate/ severe FI | 1876 | ARR 1.09 (95% CI 0.73, 1.62) |  |
| **Other neonatal morbidity outcomes** | | | | | | | |
| Tarasuk et al., 2020 [7] | Congenital anomalies | USDA HFSSM 18 items | FS | Marginal FI | 1790 | ARR 1.96 (95% CI 0.99, 3.86) | Age, partnership status, previous live birth, housing tenure, rural residence, main income source household, immigration status, race, education. |
|  |  |  | FS | Moderate/severe FI | 1876 | ARR 1.13 (95% CI 0.52, 2.45) |  |
| Cheu et al., 2020 [1] | Neonatal hypoglycaemia | USDA HFSSM 10 items | Adequate FS | Inadequate FS | 299 | COR 1.49 (95% CI 0.31, 7.06) | None |
|  | Glucose <40mg/dl in first 24hrs |  | Adequate FS | Inadequate FS | 299 | COR 0.88 (95% CI 0.19, 4.01) |  |
|  | Neonatal respiratory distress disorder |  | Adequate FS | Inadequate FS | 299 | COR 1.36 (95% CI 0.29, 6.38) |  |
|  | 5-minute Apgar score <7 |  | Adequate FS | Inadequate FS | 299 | COR 0.80 (95% CI 0.09, 6.45) |  |
| Bihan et al. 2023 [8] | Shoulder dystocia | Single item | Not FI | FI | 887 | COR 1.52 (95%CI 0.06, 37.49)* | None |
|  | Neonatal death and stillbirth | Single item | Not FI | FI | 887 | COR 0.91 (95%CI 0.04, 19.06)* | None |
|  | Neonatal hypoglycaemia | Single item | Not FI | FI | 887 | COR 0.85 (95%CI 0.24, 2.97) | None |
| Joseph et al., 2023 [12] | Stillbirth | Hunger vital sign 2 screening items | Not FI | FI | 1,065 | **ARR 2.71 (95%CI 1.13, 6.45)** | Maternal age at delivery, insurance type, and parity |

**Bold** indicates statistically significant result.  *Results included in meta-analysis. FI - Food Insecurity; FS - Food Security; MD – Mean Difference; SD - Standard Deviation; IQR – Interquartile Range; CI - Confidence Interval; OR – Odds Ratio; AOR - Adjusted Odds Ratio; COR- calculated Odds Ratio; RR- Relative Risk; ARR- Adjusted Relative Risk; SGA- Small for Gestational Age; LGA-Large for Gestational Age; NICU- Neonatal Intensive Care Unit; NR- Not reported.

Note: ^high birthweight was defined using both of the following criteria: 1) macrosomia (4000 g or more), or 2) large for gestational age (weight 90th percentile or higher by gestational age).

**References**

1. Cheu L, Yee L, Kominiarek M. Food insecurity during pregnancy and gestational weight gain. American journal of obstetrics and gynecology. 2020;220(1):204-.10.1016/j.ajog.2018.11.309.

2. Grilo SA, Earnshaw VA, Lewis JB, Stasko EC, Magriples U, Tobin J, et al. Food Matters: Food Insecurity among Pregnant Adolescents and Infant Birth Outcomes. J Appl Res Child. 2015;6(2)

3. Goin DE, Izano MA, Eick SM, Padula AM, DeMicco E, Woodruff TJ, et al. Maternal Experience of Multiple Hardships and Fetal Growth: Extending Environmental Mixtures Methodology to Social Exposures. Epidemiology. 2021;32(1):18-26.10.1097/ede.0000000000001272.

4. Eagleton SG, Shriver LH, Buehler C, Wideman L, Leerkes EM. Longitudinal Associations Among Food Insecurity During Pregnancy, Parental Mental Health Symptoms, Controlling Feeding Styles, and Infant Food Responsiveness. The Journal of Nutrition. 2022;152(12):2659-68.10.1093/jn/nxac225.

5. Sullivan K, St John M, DeFranco E, Kelly E. Food Insecurity in an Urban Pregnancy Cohort. Am J Perinatol. 2021;40(1):57-61.10.1055/s-0041-1729159.

6. Luke S. Neighborhood deprivation, food insecurity and gestational weight gain.: University of South Florida; 2017.

7. Tarasuk V, Gundersen C, Wang X, Roth DE, Urquia ML. Maternal Food Insecurity is Positively Associated with Postpartum Mental Disorders in Ontario, Canada. J Nutr. 2020;150(11):3033-40.10.1093/jn/nxaa240.

8. Bihan H, Nachtargeale C, Vicaud E, Sal M, Berkane N, Pinto S, et al. Impact of experiencing multiple vulnerabilities on fetal growth and complications in women with hyperglycemia in pregnancy. BMC Pregnancy Childbirth. 2023;23(1):740.10.1186/s12884-023-06048-9.

9. Duh-Leong C, Perrin EM, Heerman WJ, Schildcrout JS, Wallace S, Mendelsohn AL, et al. Prenatal Risks to Healthy Food Access and High Birthweight Outcomes. Acad Pediatr. 2023.10.1016/j.acap.2023.08.017.

10. Tucker CM, Berrien K, Menard MK, Herring AH, Daniels J, Rowley DL, et al. Predicting Preterm Birth Among Women Screened by North Carolina's Pregnancy Medical Home Program. Matern Child Health J. 2015;19(11):2438-52.10.1007/s10995-015-1763-5.

11. Sandoval VS, Jackson A, Saleeby E, Smith L, Schickedanz A. Associations Between Prenatal Food Insecurity and Prematurity, Pediatric Health Care Utilization, and Postnatal Social Needs. Acad Pediatr. 2021;21(3):455-61.10.1016/j.acap.2020.11.020.

12. Joseph NT, Stanhope KK, Geary F, McIntosh M, Platner MH, Wichmann HK, et al. Social Determinants of Health Needs and Perinatal Risk in Socially Vulnerable Pregnant Patients. J Health Care Poor Underserved. 2023;34(2):685-702.10.1353/hpu.2023.0058.

13. Cooper S, Graham M, Kuo CL, Khangura R, Schmidt A, Bakaysa S. The Relationship between Food Security and Gestational Diabetes among Pregnant Women. AJP Reports. 2022;12(3):E131-E8.doi:10.1055/s-0042-1751082.
