# Supplementary material for: Associations between food insecurity in high-income countries and pregnancy outcomes: A systematic review and meta-analysis
Source: PLoS Med. 2024 Sep 10;21(9):e1004450. doi: 10.1371/journal.pmed.1004450 (PMC11386426; doi:10.1371/journal.pmed.1004450)
Supplement: Attachment — Submitted filename: Reviewers comments and response.docx [file pmed.1004450.s011.docx]

Dear Dr Katrien Janin,

Thank you for previously considering our manuscript (PMEDICINE-D-23-01457R1) **“Associations between food insecurity in high-income countries and maternal physical and mental health and infant health: a systematic review and meta-analysis"** for publication in PLOS Med.

We would like to thank the editors and peer reviewers for their time to review the manuscript and for the helpful comments. We have addressed all feedback and have submitted the revised manuscript and a response to each of the editors and reviewers' comments in the table below.

We look forward to hearing from you,

On behalf of all authors,

Prof Nicola Heslehurst, Professor of Maternal and Child Nutrition, Newcastle University.

| **Editor feedback** | **Authors response** |
| --- | --- |
| 1. The authors use fixed effects models when the I2 indicates high heterogeneity. The high level of heterogeneity in the meta-analytical summary statistics can be dealt with using random effects (please also see the reviewer comment on this) | Thank you for this comment. We originally used random effects meta-analysis but based on peer reviewer feedback from the previous submission, we changed to fixed effects due to small number of studies included. As a team we have discussed fixed and random effects at length and agree that our original analysis using random effects was the most appropriate approach. We have amended the meta-analysis in this revision to use random effects models and updated the methods and results sections accordingly. |
| 2. Food insecurity will - obviously - not exist in isolation. There will be myriad associated factors. The language of causality needs to be removed given the potential for some of the associations to be explained by other factors associated with food insecurity but not fully corrected for in the analyses (i.e. residual confounding). | Thank you for this feedback on the use of language in our manuscript. We have reviewed the results section and changed our phrasing to align with this comment. |
| 3. Focus on the results where the associations are clearly positive and the 95% CI exclude unity. It is ok to mention trends but it should be accompanied by discussion of the 95% CI and the acknowledgement that the data are also consistent with no effect. | We have now decided, based on peer reviewer feedback, to remove discussion of positive trends when the 95% CIs exclude unity. |
| Requests from the editors:  1) In line with the Academic Editor and the statistical reviewer (see below), we also think the use of fixed effects models in the face of high heterogeneity is questionable. The high level of heterogeneity in the meta-analytical summary statistics can be dealt with using random effects (it could also be good to see both the FE and RE estimates). | We have changed the meta-analysis method to use random effects and updated the manuscript accordingly. |
| 2) Minor request: We wonder if perhaps it is best to more explicit about the fact that most of the studies are not just from North America, but from the US (also to be added in the Abstract as a limitation maybe?) | We have added more detail to the conclusion part of the abstract to reflect the discussion section which highlights that most studies were from the USA rather than North America more broadly. |
| Author summary: | We have added an author summary of the review findings using the guidance and places immediately following the abstract within the manuscript. |

| **Reviewer’s comments** | **Authors response** |
| --- | --- |
| **Reviewer one** | |
| Paper is much improved. | Thank you for this positive feedback and your input into this manuscript in prior rounds of reviewer’s comments. |
| **Reviewer two** | |
| The paper by Bell et al presents a systematic review and meta-analysis of observational studies looking at the association between food insecurity during pregnancy and maternal and birth outcomes. This review looks at the use of statistics in the paper.  These are generally fine. Basic meta-analysis methods are appropriately applied. Other planned analyses, using meta-regression, sensitivity analyses, and looking at publication bias, were not done, due to the relatively small number of studies involved in each meta-analysis; this is in line with general recommendations, so is acceptable. I have a few comments, though these are fairly minor. | Thank you for this positive feedback. |
| At various places in the paper, the authors lapse into language that implies a causal link between food insecurity and outcomes. For example, in the abstract, lines 36-37, "Meta-analysis showed that food insecurity significantly increased high stress level", or in the first sentence of the conclusions, "This review has identified that food insecurity has substantial impacts on maternal health and wellbeing". As with any observational research, however plausible the causal links may be, this sort of language should generally be avoided. I can tell that the authors are aware of this, from the discussion of limitations, and I know how hard it can be to get the wording right at times, but I suggest a little more editing is required. | As per the editor’s comments we have reviewed the language used throughout the manuscript to ensure that causality is not implied. |
| Similarly, I know how hard it can be to avoid the overuse of "significant" or "significantly" when reporting results. In this paper, however, the authors have a tendency to use phrases such as "non-significant positive association", which is a slight problem. If an association is not (statistically) "significant", then there is no evidence of an association. Is it then appropriate to highlight whether each non-association is positive or negative? I think I understand what the authors are trying to do; even though many associations are not statistically significant, there is a general pattern among the point estimates, with a tendency for poorer outcomes amongst those with food insecurity. I think this is ok to highlight, as part of the discussion, but flagging every estimate in the text of results section may be going a little too far. | We have reviewed the results section and changed our phrasing relating to non-significant/no association, and reviewed where most appropriate to use significant and positive association terminology. |
| Only fixed effects analyses are presented. Given the high levels of heterogeneity, and for some analyses, the large weight applied to a single study, some might argue that random effects analyses would be preferred. Perhaps both results could be presented? Otherwise, I think that more justification for the choice of only using fixed effects methods is required. | As per the editor’s comments as a team we had extensive conversations about fixed and random effects. We agree that random effects is the most appropriate approach and have amended the analysis, methods and results accordingly. |
| In the methods section, there is a reference to R Shiny being used for forwards and backwards citation chaining. I thought R Shiny was a package for building web applications. Would it be more accurate to say that citation chaining was done using an app developed in R Shiny? Or have I misunderstood? | We have amended the wording for this: We also conducted forward and backwards citation chaining for all included studies using citation chaser (an R package and Shiny app). |
| Line 372-373: why no confidence interval? | Thank you for this comment but unfortunately, we can’t find any results without a confidence interval within these lines or around these lines. |
| Line 384-385: the CI looks very asymmetrical, when I would expect it to be symmetrical for a beta coefficient. | The meta-analysis has been updated using random effects and the effect size and CIs have been amended. |
| **Reviewer three** | |
| This is a well-conducted and well-written systematic review and meta-analysis on an important and highly relevant topic (the effect of food insecurity on maternal and infant outcomes), given the cost-of-living crisis and financial pressures faced by high-income countries. It is a re-submission of a paper that I have reviewed previously, and the authors have addressed my concerns. They do a particularly good job of discussing the complexities of analysing these associations using observational data, whilst also emphasising the strength of the evidence for poor maternal and infant outcomes in affected populations. I also note that their searches have been updated to include studies up to November 2023. I recommend that the paper is suitable for publication. | Thank you for your positive feedback on our manuscript. |
| In the PROSPERO record, it states that random effects models will be used for the meta-analysis if the I² is more than 75%. However, fixed-effects models are used throughout (even with an I² of 94.6% for high stress levels). In the version of the paper that I reviewed previously, random effects models were used. The conclusions do not change between the versions (unless new studies have been added), but it would be good to add a sentence to the manuscript justifying the use of fixed-effects models and any sensitivity analyses conducted (to reassure readers that these do not change the conclusions). | We originally used random effects modelling but changed our approach based on prior reviewer feedback. We agree with your comment and as per prior editor and reviewer comments have now reverted to random effect meta-analysis. |
| In the reporting of the results, "significant" or "non-significant" are used throughout. I do not know the journal's policy on this (but know that other journals discourage the use of these terms).  I would recommend that the phrasing is changed where the confidence intervals for the pooled ratio estimates cross 1, from "non-significant positive association" (for example, in lines 279-281, "non-significant positive association between food insecurity and caesarean section (OR 1.18 95% CI 0.85, 1.64") to "no association" (or the journal's preferred phrasing). | We have reviewed the journals guidelines and were unable to find specific guidance on the use of ‘significant’ or ‘non-significant’. We have however, reviewed the results section and changed our phrasing to be more selective in using significant and positive associations where appropriate, and changed the reference to non-significant positive associations to no association. |
