# Supplementary material for: Associations between food insecurity in high-income countries and pregnancy outcomes: A systematic review and meta-analysis
Source: PLoS Med. 2024 Sep 10;21(9):e1004450. doi: 10.1371/journal.pmed.1004450 (PMC11386426; doi:10.1371/journal.pmed.1004450)
Supplement: Attachment — Submitted filename: Reviewers comments and response.docx [file pmed.1004450.s012.docx]

Dear Dr Katrien Janin,

Thank you for planning to accept our manuscript (PMEDICINE-D-23-01457R1) **“Associations between food insecurity in high-income countries and maternal physical and mental health and infant health: a systematic review and meta-analysis"** for publication in PLOS Med providing the remaining issues are addressed.

We would like to thank the editors and peer reviewers for their time to review the manuscript and for the helpful comments. We have addressed all feedback and have submitted the revised manuscript and a response to each of the editors and reviewers' comments in the table below.

We look forward to hearing from you,

On behalf of all authors,

Prof Nicola Heslehurst, Professor of Maternal and Child Nutrition, Newcastle University.

| **Editor feedback** | **Authors response** |
| --- | --- |
| 1. Thank you for providing your PRISMA checklist. When completing the checklist, please use section and paragraph numbers, rather than page numbers. (For example: Methods, paragraph 2). Please also add the following statement, or similar, to the Methods: "This study is reported as per the Preferred Reporting Items for Systematic Reviews and Meta-Analyses (PRISMA) guideline (S1 Checklist)." Feel free to add here aswell that you are also have provided a MOOSE (Meta-analyses Of Observational Studies in Epidemiology) Checklist (and add the appropriate Sx link to your manuscript). | Thank you for this feedback, we have amended the checklist to provide greater clarity for the reader on where relevant information is located within the manuscript. We have also amended the Methods section to include the statement as suggested for both PRISMA and MOOSE guidelines. |
| 2. Statistical reporting : As per standard PLOS Medical requirements, please provide 95% CIs and p values for all results where appropriate (including the abstract). We suggest reporting statistical information in the following format: ‘x’; (95% CI [‘y’,’ z’] p value) and use commas as opposed to hyphens (as these can be confused with negative values) to separate upper and lower bounds. For p values, please report as p<0.001 and where higher as 'p=0.002'. Please add the statistical method used to your method section. We also invite you to report p values to consistently to the third decimal digit - thousandths. | We have amended the formatting of reporting statistical information of CI to use square brackets throughout the entire results section and abstract. P values are reported in the correct format. |
| 3. ACKNOWLEDGMENTS/ DECLARATIONS Please remove all statements apart from acknowledgements, author contributions and abbreviations from the end of the main manuscript and include these only in the relevant parts of the manuscript submission form. Funding, competing interest, and data availability will be compiled as metadata. | Thank you for this clarification, we have removed all statements apart from acknowledgements, author contributions and abbreviations. |
| 4. Please cite the reference numbers in square brackets. Citations should precede punctuation. | References are now in square brackets. |
| 5. References: Where website addresses are cited, please specify the date of access (e.g. [accessed: 12/06/2023]). - E.g see reference 70. Please check and amend thorough. | Thank you for noticing this missing detail in the website reference. We have amended reference 70 and checked all references throughout. |
| 6. Supplementary materials: Please note that supplementary materials are not checked and will be posted as supplied by the authors. Therefore, please double check. Please cite your Supporting Information as outlined | Thank you, we have checked supporting information and how it is referred to within the manuscript. |
| Social media: To help us extend the reach of your research, if you not have already done so please provide any X (formerly known as Twitter) handle(s) that would be appropriate to tag, including your own, your co-authors’, your institution, funder, or lab. Please enter in the submission form any handles you wish to be included when we post about this paper. | We have provided our social media X handles for publication. |

| **Reviewer’s comments** | **Authors response** |
| --- | --- |
| **Reviewer two** | |
| I thanks the authors for their consideration of my original points, and I have no further comments.  One point of clarification: the missing confidence interval is now on line 399 of the revised manuscript: "mental health symptoms (β 0.54, p<0.001)". Confidence intervals are given for other beta coefficients, so I assume this is just an oversight. | Thank you for taking time to review our manuscript and informing us of this missed confidence interval. We have double checked the original paper and the authors have not reported the confidence interval for this result which is reported from a path analysis. We have clarified in the manuscript that this is a result from path analysis. |
| **Reviewer three** | |
| This is the second revision of this paper that I have reviewed. It is an important and timely systematic review. The authors have addressed the comments of all reviewers thoroughly. I recommend that the paper is published | Thank you for this positive feedback and your input into this manuscript in prior rounds of reviewer’s comments. |
